# Enterotoxigenic *Escherichia coli* infection of weaned pigs: Intestinal challenges and nutritional intervention to enhance disease resistance

**DOI:** 10.3389/fimmu.2022.885253

**Published:** 2022-08-05

**Authors:** Kwangwook Kim, Minho Song, Yanhong Liu, Peng Ji

**Affiliations:** ^1^ Department of Animal Science, University of California, Davis, Davis, CA, United States; ^2^ Division of Animal and Dairy Science, Chungnam National University, Daejeon, South Korea; ^3^ Department of Nutrition, University of California, Davis, Davis, CA, United States

**Keywords:** enterotoxigenic *Escherichia coli*, intestinal health, nutritional intervention, post-weaning diarrhea, weaned pigs

## Abstract

Enterotoxigenic *Escherichia coli* (ETEC) infection induced post-weaning diarrhea is one of the leading causes of morbidity and mortality in newly weaned pigs and one of the significant drivers for antimicrobial use in swine production. ETEC attachment to the small intestine initiates ETEC colonization and infection. The secretion of enterotoxins further disrupts intestinal barrier function and induces intestinal inflammation in weaned pigs. ETEC infection can also aggravate the intestinal microbiota dysbiosis due to weaning stress and increase the susceptibility of weaned pigs to other enteric infectious diseases, which may result in diarrhea or sudden death. Therefore, the amount of antimicrobial drugs for medical treatment purposes in major food-producing animal species is still significant. The alternative practices that may help reduce the reliance on such antimicrobial drugs and address animal health requirements are needed. Nutritional intervention in order to enhance intestinal health and the overall performance of weaned pigs is one of the most powerful practices in the antibiotic-free production system. This review summarizes the utilization of several categories of feed additives or supplements, such as direct-fed microbials, prebiotics, phytochemicals, lysozyme, and micro minerals in newly weaned pigs. The current understanding of these candidates on intestinal health and disease resistance of pigs under ETEC infection are particularly discussed, which may inspire more research on the development of alternative practices to support food-producing animals.

## Introduction

Modern swine production becomes highly intensive in order to maximize productivity, however, husbandry-associated stress is also increased. Many physical and/or psychological stress, such as environmental and nutritional changes and increased exposure to infectious diseases, can induce a significant depression in growth performance, alter local or systemic immune responses, and disrupt gastrointestinal homeostasis in different physiological stages of pigs (1). For instance, regrouping, crowding, social isolation, and maternal deprivation may impair the immunity and alter the regulation of the neuroendocrine in pigs, thus inducing gastrointestinal diseases (2). This review is mainly focused on newly weaned pigs and post-weaning stress. The development of the intestinal epithelial barrier, immunity, and enteric nervous system exhibits a high degree of plasticity in the post-weaning period, which can impact the long-term phenotypes and gastrointestinal function (3, 4). Infectious diarrhea disease has long been one of the leading causes of morbidity and mortality in the swine industry (5). Post-weaning diarrhea (PWD) induced by pathogenic Escherichia coli (*E. coli*) infection is one of the most common diseases and is characterized by the discharge of watery feces, dehydration, a thin or unthrifty appearance, and sudden death of piglets. During acute outbreaks of PWD, the pig mortality due to *E. coli* infection may reach 20 to 30% over a 1- to 2-month time span among infected pigs (6). A survey conducted by National Animal Health Monitoring System reported that the mortality rate of nursery pigs ranged from 2.6 to 3.6% in the years 2000, 2006, and 2012, of which diarrhea-caused deaths accounted for 9.4 to 12.6% of the overall mortality (5). Therefore, the prevention of post-weaning *E. coli* infection is extremely important to maintain growth performance and welfare of pigs during the entire lifespan. In-feed antibiotics and a number of feed additives/supplements are discussed in this review to summarize their efficacies on growth promotion and disease resistance in weaned pigs.

## ETEC infections in weaned pigs

The commensal *E. coli* strains that colonize the gastrointestinal tract of pigs rarely cause disease. However, *E. coli* expressing specific virulence features confers the ability to cause diarrheal disease (7). The major pathotypes of *E. coli* include enteropathogenic *E. coli* (EPEC), enterohaemorrhagic *E. coli* (EHEC), enteroaggregative *E. coli* (EAEC), enteroinvasive *E. coli* (EIEC), diffusely adherent *E. coli* (DAEC), Vero- or Shiga-like toxin-producing *E. coli* (VTEC or STEC) and enterotoxigenic *E. coli* (ETEC) (8). Among these, the most diffuse etiological agents responsible for PWD in pigs are ETEC displaying the fimbriae F4 (K88) and F18 (9).

### Clinical signs

The clinical signs observed in *E. coli*-infected animals include diarrhea with watery, light-orange-colored feces, loss of appetite (decreased feed intake), depression, dehydration, rough hair coating, and inflamed perianal regions smeared with feces (10). The watery diarrhea condition typically lasts from 1 to 5 days after infection, but severe cases may result in shock or sudden death without showing obvious symptoms of illness (11). Pigs also change in appearance, as a sallow discoloration of the tip of the nose, the ears, and the abdomen may be observed.

### Pathogenesis and toxins

**Figure 1:**
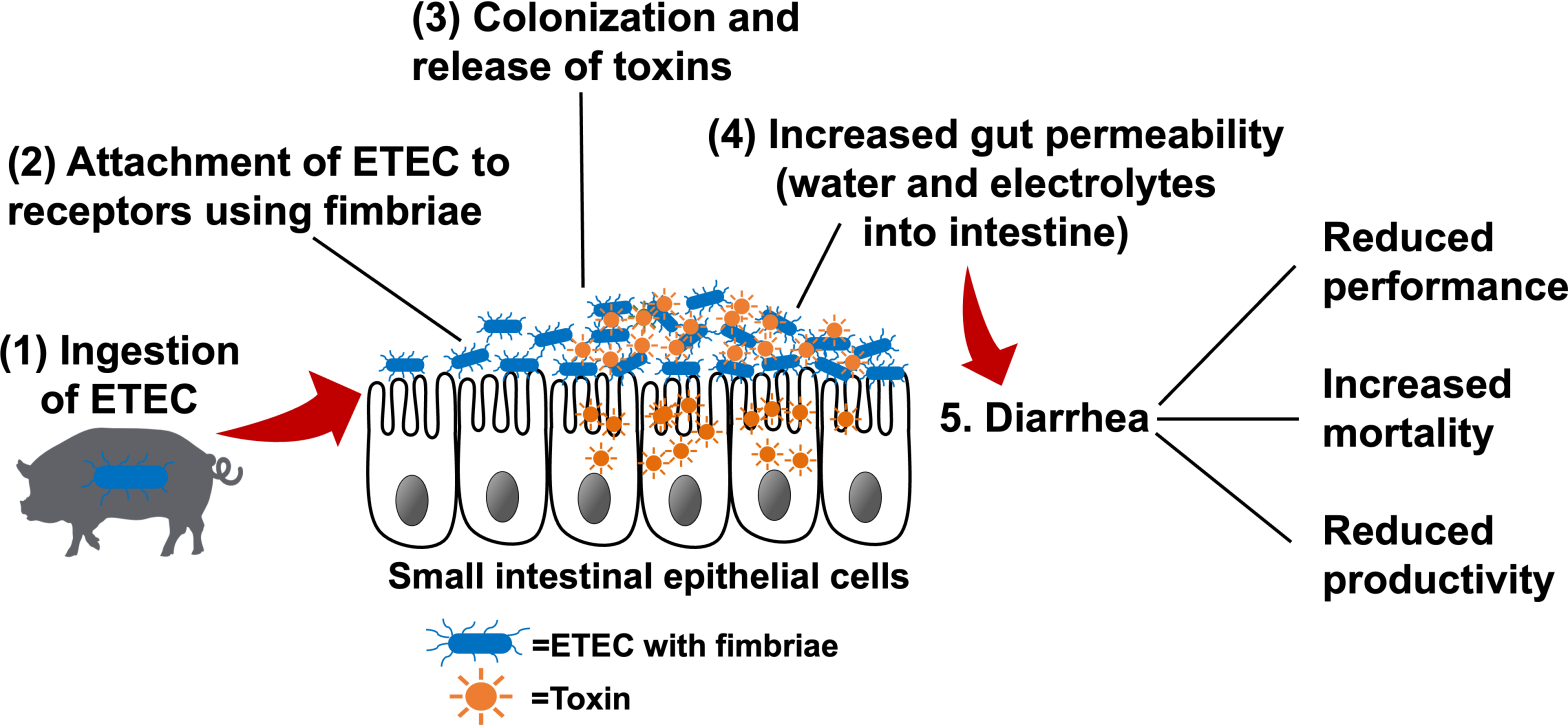
The pathogenesis of enterotoxigenic *Escherichia coli* (ETEC**)** (1) ETEC are ingested by susceptible pigs and enter the gastrointestinal tract. (2) ETEC express fimbrial adhesins, which mediate adherence to specific receptors present on the intestinal epithelial cells. (3) Bacterial colonization occurs in the small intestinal mucosa. Once colonization is established, ETEC rapidly produce toxins (e.g., heat-labile, heat-stable, and/or Shiga toxins). (4) Enterotoxins stimulate water and electrolyte loss into the intestinal lumen, increase gut permeability, and/or transport across the epithelial cells to blood circulation, resulting in edema. (5) Increased gut permeability and massive water loss into the intestinal lumen lead to diarrhea, which results in the poor performance and productivity and increased mortality. Adapted from: Nagy and Feteke (12), Kaper et al. (13), Croxen et al. (14), Fleckenstein et al. (15), and Mirhoseini et al. (16), Rhouma et al. (17).

The pathogenesis of ETEC-induced diarrhea is initiated by bacterial attachment to specific receptors expressed on the intestinal epithelium, followed by colonization of ETEC in the small intestine (**Figure 1**) (18). Fimbriae are hair-like appendages that show characteristic patterns from the outer membrane of the bacterial cells, which facilitate the adhesion of ETEC to the small intestinal mucosa (19). In pigs, F4 and F18 are the fimbrial types that are mostly associated with PWD, and these two fimbrial genes were found in 92.7% of all ETEC-induced PWD (20). Once ETEC successfully adheres to the small intestinal epithelium, colonization is established, and ETEC rapidly proliferates to produce one or more types of enterotoxins.

The secreted enterotoxins, including heat-labile toxins (LTs) and heat-stable toxins (STs), act on stimulating water and electrolyte secretion and reduce fluid absorption in the small intestine (12). Briefly, LTs bind to the receptors on the cell surfaces and activate the adenylate cyclase system to stimulate the secretion of cyclic adenosine monophosphate (cAMP). The up-regulated cAMP induces the activation of an apical chloride channel and a basolateral Na/K/2Cl cotransporter, resulting in chloride secretion from the apical region of enterocytes, reduced sodium absorption, and a concomitant massive water loss into the intestinal lumen (21, 22). STs produced by ETEC are secreted peptides that can be classified as STa and STb based on their solubility and enzyme sensitivity. STa binds to the extracellular domain and accumulates cyclic guanosine monophosphate (cGMP) and consequently opens the ion channel to induce Cl^-^ and HCO_3_^-^ release into the intestinal lumen (23). STa also enhances the luminal secretion of pro-inflammatory cytokines and chemokines, including interleukin (IL)-6 and IL-8, in the small intestinal mucosa of pigs (24). STb was shown to specifically interact with calcium ion channels on the intestinal epithelial surface to elevate intracellular Ca^2+^ concentration, which may increase paracellular permeability *via* claudin-1 redistribution (25). STb can also concurrently reduce the expression of other tight junction proteins, including zona occludens-1 and occludin, thus accelerating fluid loss into the intestinal lumen (26).

Moreover, Shiga toxins (Stxs) and lipopolysaccharides (LPS) derived from ETEC are also involved in the pathogenicity in the host (13). After binding to the cell surface, Stxs are internalized *via* the Golgi apparatus to the endoplasmic reticulum before being translocated to the cytosol of enterocytes (27). During the translocation, Stxs are able to induce DNA fragmentation and cell apoptosis of infected cells, which further facilitates proteolysis in neighboring cells and toxic effects on the host (28). Additionally, Stxs can stimulate the intestinal epithelial cells to secrete pro-inflammatory cytokines and neutrophil chemoattractant molecules, like IL-8 (29). Bacterial LPS are the major components of the outer membrane of Gram-negative bacteria, including ETEC (30). LPS receptors are mainly located on the cells in the innate immune system, such as macrophages and endothelial cells (31). The activation of immune cells induced by LPS binding can stimulate various immunological signaling pathways, leading to the release of a large amount of cytokines, including tumor necrosis factor-α (TNF-α), IL-6, and IL-1 from target cells (32).

## Intestinal barrier disruption during ETEC infection

The intestinal epithelium forms as a single layer lining the gastrointestinal tract and is responsible for the uptake of nutrients and water. Meanwhile, the epithelium also serves as a physical barrier to exclude potential antigens, pathogens, and toxins from the external environment (33). ETEC infection could damage the intestinal epithelial barrier functions, resulting in electrolytes and water imbalance and watery diarrhea, and induce intestinal inflammation in piglets (19, 34, 35).

### Mucus

The mucus layer is a gel-like sieve structure covering the luminal surface of the gastrointestinal tract and acts as a physical barrier to bacteria and other antigens present in the lumen (36, 37). Mucus is known to be a highly dynamic matrix, mainly consisting of glycosylated mucin proteins secreted by intestinal goblet cells. In the small and large intestine, mucin 2 (MUC2) is the most abundant mucus protein (38). However, the inner mucus layer also contains antimicrobial peptides, immunoglobulin-A (IgA), and other molecules that are essential in the innate immune defense and the maintenance of intestinal homeostasis (39).

ETEC infection could alter the expression of *MUC2* in the small intestine. An *in vivo* ETEC F18 challenged study observed that ETEC-infected pigs expressed more *MUC2* gene in the jejunal mucosa during the peak of infection (40). The up-regulated *MUC2* gene in the intestinal epithelium was also reported by several *in vitro* studies when LTs or Stxs producing ETEC were used (41, 42). However, a down-regulated *MUC2* gene expression was observed in ETEC F18 infected pigs during the post-peak infection period (40). A growing body of evidence demonstrated that a highly conserved mucin-degrading metalloprotease from ETEC is responsible for mucin reduction, which facilitates the interaction of ETEC with intestinal enterocytes and immune cells and triggers inflammatory responses in the gut (43–45).

### Tight junction and epithelial barrier

Intestinal tight junctions are junctional complex in epithelium, consisting of three integral transmembrane proteins, including occludin, claudins, and junctional adhesion molecule (JAM), as well as the cytoplasmatic plaque proteins zonula occludens, cingulin, and 7H6 (46, 47). Occludins and claudins are the major sealing protein. Zonula occludens directly interact with most of the transmembrane proteins localizing at tight junctions and provide the structural basis for the assembly of multiprotein complexes at the cytoplasmic surface of intercellular junctions (48, 49). Tight junctions act as gates or fences to control intestinal permeability and to maintain intestinal integrity (50). Beyond that, growing evidence indicates that tight junctions are also involved in cell-cell signal transduction to guide cell proliferation and differentiation (51).

The alteration of tight junction proteins by bacterial pathogens or enterotoxins can lead to permeability defects in the intestinal epithelium (52). Numerous research articles have reported that ETEC can impair intestinal barrier function by modulating tight junction protein expressions (**Table 1**), which may induce diarrhea and initiate inflammatory cascades. The common methods to assess *in vivo* intestinal permeability include the mannitol and lactulose test, analyzing the flux of intact FD4, and measuring bacterial translocation. The flux of intact FD4 across the intestinal epithelium occurs primarily through paracellular pathways, thus, increased flux rates of FD4 can reflect the intestinal barrier defects (62). McLamb et al. (34) and Kim et al. (40) reported that ETEC F18 infection elevated FD4 flux rates across the porcine ileum or jejunum, respectively. Bacterial translocation is defined as the passage of viable bacteria or its products from the gastrointestinal tract to normally sterile tissues, including mesenteric lymph nodes and other internal organs (i.e., the spleen) (63, 64). The major mechanisms promoting bacterial translocation are intestinal bacterial overgrowth, deficiencies in host immune defenses by disturbed gut integrity, and increased permeability or mucosal injury (65). It was reported that ETEC F18 clearly increased bacterial translocation from the intestinal lumen to the mesenteric lymph nodes of weaned pigs (66). Collectively, ETEC infection negatively impacts tight junction integrity, and increases paracellular movements of molecules, thus inducing inflammatory responses and diarrhea in pigs.

**Table 1:** Enterotoxigenic *Escherichia coli* (ETEC) altered the expression of tight junction proteins in the small intestine of pigs *in vivo* or epithelial cells *in vitro*.

Pathogen	Pig age	Tight junction proteins/outcomes	Reference
ETEC K88	35 d	Reduced protein expression of *occludin* and *claudin* in ileum	Ewaschuk et al. (53)
ETEC K88		Altered the distribution of *ZO-1* and *claudin* in porcine Caco-2 cells (fluorescence microscopy analysis)	Yu et al. (54)
ETEC K88	35 d	Reduced mRNA expression of *occludin* in jejunum; reduced expression of *ZO-1* in jejunum and ileum	Gao et al. (55)
ETEC K88	18 d	Reduced mRNA expression of *occludin* in jejunum	Yang et al. (56)
ETEC K88		Reduced mRNA expression of *ZO-1*, *claudin*, and *occludin* in porcine IPEC-J2 cells; Reduced protein expression of *claudin* and *occludin*g in porcine IPEC-J2 cells	Wu et al. (57)
ETEC K88	36 d	Reduced protein expression of *ZO-1* and *occludin* in jejunum	Yang et al. (58)
ETEC K88	36 d	Reduced protein expression of *ZO-1* in jejunum	Li et al. (59)
ETEC F18	39 d	Reduced mRNA expression of *claudin* in jejunum	Kim et al. (40)
ETEC F18	37 d	Reduced mRNA expression of *claudin* in ileum	Li et al. (60)
ETEC F18	38 d	Reduced mRNA expression of *ZO-1* and *occludin* in ileum	Becker et al. (61)

## Immune responses of pigs during ETEC infection

In addition to the physical barrier function of the mucus, the mucosal immune system constitutes an extensive and highly specialized innate and adaptive immune system to protect the host against potential insults from the environment (67). When inflammation occurs in the intestines, the robust innate immune responses are first observed by the marked elevations in the production of inflammatory mediators, including IL-1β, IL-6, and IL-8, which further promote leukocyte accumulation and survival in the inflamed sites (68). The recruited neutrophils and activated macrophages are responsible for the elimination of pathogens and stimulating systemic inflammation and acute-phase reaction (69, 70). ETEC F18 that expressed LT, STa, and Shiga-like toxins remarkably induced the recruitment of neutrophils and macrophages in the ileum of weaned pigs during the peak of infection (71). Consistently, the up-regulated expression of genes encoding inflammatory mediators (e.g., *COX2*, *IL1B*, *IL6*, *IL7*, and *TNF*) were also observed in the ileal mucosa of ETEC F18 infected piglets (40). LPS, the major component of the outer member of ETEC, are highly involved in the activation of innate immunity, as indicated that ETEC F18 increased the mRNA expression of LPS binding protein and *MyD88* in ileal mucosa of pigs (72). In addition, flagellin, a globular protein in the flagella of ETEC, is also involved in the activation of intestinal immune responses by stimulating IL8 expression in the ileal mucosa (72, 73). Several pathways may be involved in the process of ETEC infection, as the NF-κB and MAPK pathways are particularly important to stimulate downstream inflammatory responses (72, 74–77).

Systemic inflammation could be evoked due to Gram-negative sepsis (78, 79). ETEC F18 infection could induce systemic inflammation, as indicated by the gradually increased total white blood cell counts, neutrophils, and lymphocytes in the blood circulation of infected pigs (71, 80, 81). Systemic levels of pro-inflammatory cytokines (e.g., TNF-α) and acute phase proteins (C-reactive protein and haptoglobin) were also elevated accordingly after ETEC F18 or K88 infection (71, 82–84). The peak of systemic inflammation in weaned pigs appears on day 2 to 7 post-ETEC challenge depending on the severity of infection, while it usually disappears or cannot be detected after day 14 post-infection.

## Intestinal microbiota changes during ETEC infection

Intestinal microbiota plays pivotal roles in maintaining the nutritional, physiological, and immunological function of the intestine (85, 86). The pig intestine harbors a very complex and diverse microbial community, which shifts along the intestinal tract and changes by age, diet, and many other factors (87). The colonization of intestinal microbiota in pigs is initiated at birth but still develops at the weaning stage (88). Thus, the intestinal microbial composition in newly weaned pigs could be easily disrupted due to weaning stress and dietary changes, making the pigs more susceptible to pathogenic bacteria (89).

Although *E. coli* is one of the first bacteria to colonize in the intestine of piglets at birth, it is phased out after weaning (90). ETEC infection or increased abundance of *E. coli* during the post-weaning stage could impact intestinal microbiota. Bin et al. (91) reported that ETEC K88 infection reduced microbial diversity and the Bacteroidetes : Firmicutes ratio in jejunum and feces in weaned pigs. Bacteroidetes and Firmicutes are the most dominant intestinal microbial phyla in young pigs, which cooperatively utilize carbohydrates in the gut (92). The reduced fecal Bacteroidetes : Firmicutes ratio is used as a biomarker for intestinal dysbiosis and was also observed in pigs with other types of diarrheal diseases (93, 94). Significant changes in community structure were also reported in many ETEC F18 or K88 infection cases. Pigs challenged with ETEC F18 or K88 were reported to have increased relative abundance of Proteobacteria family in the ileum or colon by increasing *Escherichia-Shigella* or *Helicobacteraceae* (91, 95–97). A reduced relative abundance of *Lactobacillus* was observed in the ileum of weaned pigs when challenged with ETEC F18 (97). The disturbance of intestinal microbiota by ETEC infection further shifts the intestinal ecosystem to be more favorable for the growth of pathogens and reduces the production of volatile fatty acids in the large intestine (97–99). Many of these microbiota changes were reported to be negatively correlated with growth performance and the overall intestinal health of weaned pigs (100, 101).

## In-feed antibiotics

In-feed antibiotics were one of the most powerful substances to prevent and treat bacterial infections in food-producing animals. In swine production, the use of antibiotics at intermediate or therapeutic levels served many purposes, including 1) treating sick animals, 2) preventing diseases by mass treatment of the entire population, 3) reducing the negative impacts of stresses, namely weaning stress, and 4) promoting growth. The potential mechanisms of action of antibiotics target different anatomical parts of bacteria. First, antibiotics could induce a lethal malfunctioning of the bacterial cell wall synthesis. The presence of penicillin-binding proteins (PBPs) is critical for proper bacterial cell wall assembly (102). However, PBPs are the main targets of β-lactam and glycopeptide antibiotics in order to inhibit bacterial cell wall synthesis. More specifically, β-lactam agents target PBPs, and their interaction could lead to failure in the synthesis of new peptidoglycan and lysis of bacterium (103). Second, antibiotics could inhibit protein biosynthesis in the ribosomes of bacterial cells. The bacterial ribosome (70S) is composed of two ribonucleoprotein subunits, 30S and 50S subunits, with each performing different functions (104). Some antimicrobial agents, like aminoglycosides and tetracyclines, target the 30S subunit by either preventing the binding of the mRNA to the ribosome or inducing misreading and premature termination of translation of mRNA (105). Chloramphenicol, macrolides, and oxazolidinones antibiotics are the major inhibitors of the 50S subunit. They can prevent the binding of aminoacyl-tRNA to the mRNA-ribosome complex or inhibit the formation of complete peptide chains by targeting the conserved sequences of the peptidyl transferase (106). Third, antibiotics can inhibit bacterial DNA replication. Quinolone antibiotics are the major DNA replication inhibitors. They can inhibit bacterial nucleic acid synthesis by disrupting topoisomerase and DNA gyrase, two critical bacterial enzymes that regulate the chromosomal supercoiling required for DNA synthesis (107). The disturbance of these enzymes can break bacterial chromosomes and cause rapid bacterial death (108). Fourth, antibiotics can inhibit folic acid metabolism. Folate is a cofactor for many enzymes that are required for DNA and RNA biosynthesis and amino acid metabolism in bacteria (109). Sulfonamides and trimethoprim interrupt folic acid synthesis and ultimately disturb the synthesis of purines and thus DNA biosynthesis (110). A combination of sulfonamides and trimethoprim has shown synergistic antibiotic activities because they target distinct steps in folic acid metabolism (111).

As reviewed by Cromwell (112), in-feed antibiotics improved growth rate by an average of 16.4% and improved the efficiency of feed utilization by 6.9% of young pigs from 7 to 25 kg body weight. Moreover, the inclusion of antibiotics in feed dramatically reduced the mortality of young pigs (3.1%) under high-disease conditions and environmental stress when comparing with non-antibiotic-treated pigs (15.6%) (113). Therefore, approximately 70% of the swine farms in the United States used a wide variety of antibiotics in nursery diets over the past 20 to 30 years before 2015 (5). Several commonly used antibiotics and their effects are summarized in **Table 2**. However, the potential risks of antibiotic resistance and contamination and the adverse health effects of trace amounts of antibiotics in humans and animals have been increasingly recognized as global health concerns (124). Therefore, effective alternative practices to strengthen the disease resistance of animals are greatly needed.

**Table 2:** In-feed antibiotics on ETEC infection of weaned pigs.

Pathogen	Antibiotics	Outcome	Reference
ETEC K88	Chlortetracycline+ sulfamethazine+ penicillin	Enhanced immunological responses, improved intestinal morphology	Nyachoti et al. (114)
ETEC K88	Colistin sulfate+ olaquindox	Enhanced feed efficiency, reduced diarrhea	Pan et al. (115)
ETEC K88	Apramycin+ tiamulin+ sulfathiazole+ bacitracin methylene disalicylate	Enhanced growth performance, improved systemic immune responses	Lee et al. (116)
ETEC K88	Colistin	Reduced mortality, reduced diarrhea	Trevisi et al. (117)
ETEC K88	Apramycin	Reduced fecal shedding of ETEC	Kim et al. (118)
ETEC K88	Carbadox	Enhanced growth performance, reduced fecal shedding of ETEC	Owusu-Asiedu et al. (119)
ETEC F18	Chlortetracycline+ tiamulin	Reduced diarrhea, improved systemic immune responses	Hong et al. (120)
ETEC F18	Carbadox	Enhanced growth performance, reduced diarrhea, enhanced intestinal integrity	He et al. (121) Kim et al. (122) Kim et al. (123)

## Nutritional intervention

Research on exploring alternatives to antibiotics is growing and has been reviewed by Pettigrew (125), Lallès et al. (126), Heo et al. (127), and Liu et al. (128). Many nutritional interventions have been widely applied to weanling pigs to enhance their disease resistance and growth performance. Although their exact protective mechanisms may vary and are still not completely understood, one or more following functions may be involved: (1) to favorably affect the characteristics of feed (2), to satisfy the nutritional needs of animals without any adverse effects, or (3) to favorably impact animal production and performance, particularly by regulating gut microbiota, intestinal immunity or digestibility of nutrients.

### Zinc oxide

Zinc is an essential micro-mineral required in trace amounts in animal feed. Zinc performs broad types of functional roles, including (1) structural roles in forming components of organs and tissues, (2) physiological roles in maintaining homeostasis, (3) catalytic roles in regulating enzymes and endocrine systems, and (4) regulatory roles in cellular replication and differentiation (129). Zinc also plays a central role in the immune system, as it is crucial for the development and function of immune cells, the production or biological activity of cytokines, and the regulation of T and B cell signaling (130–132). Zinc deficiency affects many aspects of innate and adaptive immunity. Acute zinc deficiency can cause decreased innate and adaptive immune responses, while chronic zinc deficiency is highly associated with many diseases and inflammation (133).

Zinc is commonly added to the nursery diet at pharmacological levels to promote performance and control post-weaning diarrhea (134–136). Numerous studies also reported that supplementation of a high dose of zinc in the form of zinc oxide (ZnO) enhanced disease resistance of weaned pigs against ETEC infection. For instance, the inclusion of 2,880 mg/kg of ZnO reduced the incidence of diarrhea and boosted the recovery of pigs from ETEC F4 infection (119). Kim et al. (118) also reported that 2,400 mg/kg of ZnO administration enhanced average daily gain, reduced diarrhea and fecal shedding of *E. coli*, and improved small intestinal morphology in weaned pigs challenged with ETEC F4. Other beneficial effects of pharmacological zinc included the enhancement of intestinal integrity (137), restoration of the injured intestinal mucosa (138), reduction of intestinal permeability by enhancing the expression of tight junction proteins (139), and improvement of intestinal immunity (140) in ETEC-infected pigs. The potential mechanisms of action of high dose ZnO in reducing post-weaning diarrhea include but are not limited to: 1) inhibiting pathogen viability and 2) modulating the intestinal microbial population. Roselli et al. (141) demonstrated that *in vitro* ZnO treatment may protect intestinal epithelial cells from ETEC F4 infection by inhibiting the adhesion and internalization of bacteria. Supplementation of 2,500 mg/kg of ZnO *in vivo* helped to stabilize the microbial community while preventing pathogenic microbes proliferation during the first 2 weeks of post-weaning (142).

Although pharmacological ZnO is very effective in preventing post-weaning diarrhea, its environmental impact is significant and increases public health and safety concerns. Recent research demonstrated that supplementation of pharmacological ZnO may induce the excessive accumulation of zinc in animal tissues, including kidney, liver, and pancreas (143, 144). The overload of ZnO might also contribute to the acquisition and spread of antibiotic resistance genes in pigs (145–147). Therefore, the use of pharmacological ZnO in piglet diets was banned in the European Union from June 2022.

### Direct-fed microbials

Direct-fed microbials (DFM) are live microorganisms that confer a health benefit on the host, when administered in adequate amounts (148). There are 3 main categories of DFM, including *Bacillus* (Gram-positive spore-forming bacteria), lactic acid-producing bacteria (e.g., *Lactobacillus*, *Bifidobacterium*, *Enterococcus*, etc.), and yeast (149). The beneficial effects of DFM on the host may be attributed to several mechanisms, including but not limited to: (1) production of antimicrobial products, (2) regulation of gut microbial profile, (3) immunomodulation, and (4) enhancement of epithelial gut barrier function (150, 151).

*Bacillus*-based DFMs are spore-forming bacteria. They are thermostable for feed storage and processing (e.g., pelleting and extrusion) and are able to survive at low pH in the stomach (152). Some common species of *Bacillus* include *Bacillus subtilis*, *Bacillus licheniformis*, *Bacillus pumilus*, *Bacillus amyloliquefaciens*, *Bacillus anthracis*, and *Bacillus cereus*, in which *Bacillus anthracis* and *Bacillus cereus* are known to be pathogenic to humans and animals (153). *Bacillus* spp. can be isolated extensively from plants and their rhizosphere (soil in the vicinity of plant roots) and can also be found in other environments (154). *Bacillus* spp. were characterized as mesophilic and neutrophilic bacteria that can survive and germinate in the gut, form biofilms, and secrete antimicrobials (155, 156). A variety of *Bacillus*-based supplements have been found to promote growth, feed utilization, and intestinal health of pigs (157–159). The potential mechanisms of action of *Bacillus* spp. against ETEC infection include: 1) modulating the host immune responses by regulating the expression of major cytokines that are involved in initiating and regulating immune responses (160), 2) enhancing the expression of tight junction proteins (161), and 3) and promoting the growth of beneficial microbes and overall gut health of the host (162). Our previously published research reported that dietary supplementation of 2.56 × 10^9^ CFU/kg of *Bacillus subtilis* (DSM 25841) enhanced disease resistance and growth performance and reduced diarrhea of weaned pigs infected with ETEC F18 (40, 121). Pigs fed with *Bacillus subtilis* also strengthened intestinal integrity and barrier function, as indicated by reduced transcellular and paracellular permeability and enhanced gene expression of tight junction protein, *ZO1*. In addition, the same *Bacillus subtilis* (DSM 25841) strain was able to reduce the incidence and severity of diarrhea in weaned pigs infected with ETEC F4 (163). Supplementation of *Bacillus subtilis* DSM 25841 was also observed to reduce cecal *Enterobacteriaceae* level, up-regulate the expression of gene sets related to immunity, and improve amino acids metabolism and utilization in jejunal mucosa (163).

Lactic acid-producing bacteria administration can modulate intestinal microbial profiles by competing for the binding sites on the intestinal epithelial cells with pathogens, or by producing microbicidal substances that inhibit or kill pathogens (164–166). *Lactobacillus plantarum* is a widespread strain that can be produced by plant fermentation or directly isolated from the gastrointestinal tract of healthy humans or animals. Lee et al. (116) and Yang et al. (56) demonstrated that supplementation of *Lactobacillus planatrum* (10^10^ cfu/kg of CJLP243 or 5 × 10^10^ cfu/kg of CGMCC 1258, respectively) enhanced growth performance and reduced diarrhea of weaned pigs challenged with ETEC F4. Pigs fed with *Lactobacillus planatrum* also had enhanced intestinal morphology, reduced fecal shedding of ETEC, or reduced adhesion of ETEC to the small intestinal mucosa. Another study reported that *Lactobacillus plantarum* (CCFM1143 or FGDLZ1M5; 5 × 10^10^ cfu/kg, respectively) supplementation reduced the relative abundance of *Bacteroidetes* and *Enterobacteriaceae* in feces and increased the concentration of total short-chain fatty acids in the cecum of ETEC infected pigs (99). Consistently, *Lactobacillus rhamnosus* (ACTT 7469; 10^10^ cfu/day or 10^12^ cfu/day) administration ameliorated ETEC F4-induced diarrhea and reduced pathogenic coliform shedding in feces, possibly due to its ability to increase the number of *Lactobacilli* and *Bifidobacteria* in feces (167). Some research also reported that supplementation of lactic acid-producing bacteria could regulate intestinal mucosa immunity and stimulate the immune system of the host (168, 169). A previous *in vitro* study reported that *Lactobacillus planatrum* (299v) could increase the mRNA expression of *MUC2* and *MUC3* in HT 29 intestinal cells, thus, inhibiting the adherence of enteropathogenic *E. coli* to the intestinal cells (170). Moreover, lactic acid-producing bacteria contribute to an acidic environment in the gastrointestinal tract, which partly alters the growth of pathogenic microorganisms, including *E. coli* (171). Therefore, lactic acid-producing bacteria is another common type of DFMs used in weaned pigs to promote intestinal health.

Yeast consists of a broad range of products, including whole live yeast cells, heat-treated yeast cells, ground yeast cells, purified yeast cell cultures, and yeast extracts. The efficacy of yeast-based products varies depending on their forms (128). The majority of the dry weight of the yeast cell wall is polysaccharides, with α-D-mannan and β-D-glucan as the major components. These polysaccharides have been recognized for their immune-regulatory activities through specific interactions with different immunocompetent cells (172). In particular, α-D-mannan in yeast was reported to bind to mannose-specific receptors that are present in many pathogenic bacteria, including *E. coli* and *Salmonella* spp., thus, inhibiting the adhesion of these pathogens to the mannose-rich glycoproteins lining the intestinal lumen (173). Growing evidence supports the immunostimulatory benefits of β-D-glucans, as it could stimulate the activity of macrophages and neutrophils *via* binding to their receptors (174). *Saccharomyces* spp. are the most studied yeast species for controlling intestinal disorders in young animals due to their remarkable immune-regulatory properties (175, 176). The beneficial effects of live *Saccharomyces cerevisiae* yeast on controlling diarrhea and reducing mortality of weaned pigs infected with ETEC F4 were reported by Trevisi et al. (117). The results of gene expression profiles in jejunal mucosa indicated that supplementation of *Saccharomyces cerevisiae* yeast modified the expression of genes related to mitosis, mitochondria development, metabolic process, and transcription in ETEC-infected pigs (177).

### Prebiotics

Prebiotics were originally defined as ‘non-digestible food substances that selectively stimulate the growth of favorable species of bacteria in the gut, thereby benefitting the host’ by Gibson and Roberfroid (178). This definition has been expanded by including three broad criteria: (1) resistance to gastric acid and hydrolysis by mammalian enzymes and gastrointestinal absorption; (2) ability to be utilized by the gastrointestinal microbiota; and (3) selectively stimulate the growth and/or the activity of intestinal bacteria associated with health-promoting effects (179). The best-characterized prebiotics are non-digestible oligosaccharides, including inulin, lactulose, pyrodextrins, fructo-oligosaccharides (FOS), galacto-oligosaccharides (GOS), xylo-oligosaccharides, transgalactooligosaccharides, and isomalto-oligosaccharides (180).

The most striking effect of prebiotics is their ability to reshape the composition of gut microbiota in the host. Prebiotics can boost the production of health-promoting bacteria, such as lactic acid-producing bacteria, which can further inhibit the growth of enteric pathogens (e.g., *E. coli*, *Campylobacter*, *Salmonella* spp.) and/or attenuate their virulence (181, 182). The enhancement of the beneficial bacteria population by adding prebiotics could indirectly affect the immunity of the host. In addition to that, prebiotics *per se* can directly interact with intestinal cells, including epithelial cells, goblet cells, or immune cells (183, 184). This interaction may trigger the downstream benefits, as indicated by more mucin production (185), strengthened gut barrier functions (186), or enhanced inflammatory responses (187, 188). Many studies have confirmed the beneficial effects of prebiotic supplementation in weaned pigs challenged with ETEC. For example, supplementation of 2.5 g/kg of FOS extracted from plants can improve growth performance and gut health of pigs infected with ETEC F4 (189). Specifically, pigs supplemented with FOS reduced plasma IL-1β and TNF-α, and improved small intestinal morphology against ETEC F4. Moreover, FOS administration also elevated mRNA expression of duodenal and jejunal *ZO-1* and ileal *occludin*, but down-regulated *TNF-α* and *IL-6* in the small intestine. These results indicated that supplementation of FOS was associated with suppressed inflammatory responses and improved intestinal barrier functions. Luo et al. (190) also observed that dietary supplementation of FOS attenuated the intestinal mucosa disruption in ETEC-infected pigs by increasing their anti-oxidative capacity and intestinal barrier functions. GOS, one of the main bioactive compounds in human milk, was well studied in humans as it supports the colonic health of breast-fed infants (191). GOS exhibited *in vitro* antimicrobial effects on ETEC F4 by inhibiting the adherence of the F4 strains to porcine intestinal mucins (192). This observation suggests that GOS may serve in the prophylaxis of ETEC infection. β-glucans originated from different sources (cereal grains, yeast, or algae) also show prebiotic properties (193). Stuyven et al. (194) reported that β-glucans extracted from yeast reduced the colonization of ETEC F4 to the small intestine, thus alleviating diarrhea of weaned pigs. However, the immune-modulatory activity of β-glucans was more attractive and well-studied in humans and animals. Our previously published research observed that supplementation of algae-derived β-glucans enhanced gut integrity, reduced intestinal paracellular permeability, and boosted intestinal and systemic immune responses in weaned pigs infected with ETEC F18 (81). This study also suggests that dectin, a major β-glucan receptor expressed on many immune cells (e.g., macrophages), is potentially involved in the immune-regulatory effects of β-glucans, thus protecting the host from the ETEC infection (81, 195).

### Phytochemicals

Phytochemicals include a large variety of secondary plant metabolites that are naturally derived from plant materials or directly synthesized (e.g., polyphenols, terpenoids, carotenoids, limonoids, flavonoids, catechins, anthocyanidins, indoles, ethnobotanicals, etc.) (196). Phytochemicals exhibited broad biological properties, including antimicrobial, antioxidant, anti-inflammatory, and antiviral effects (197–200). Notably, many phytochemicals display broad-spectrum antibacterial activities against Gram-negative and Gram-positive bacteria (201–203). The antimicrobial mechanism of action varies due to the sources and extraction methods of phytochemicals. Based on the literature view, several potential antimicrobial mechanisms were proposed. First, many plant-derived essential oils could destabilize the phospholipid bilayer, causing the loss of permeability, leakage of intracellular constituents (e.g., ions, proton), and even the coagulation of cytoplasm (204, 205). Second, some phytochemicals contain a high proportion of phenolic compounds that possess strong antibacterial properties by inhibiting the efflux pump (206). Third, phytochemicals could disrupt the enzymes involved in the synthesis, replication, repair, and transcription procedures of virulent bacteria (207). Fourth, certain active components in phytochemicals may prevent the development of adhesion formation (208, 209) and inhibit bacterial adhesion (210, 211).

The anti-inflammatory effects of phytochemicals have also been widely reported with *in vitro* and *in vivo* models. For example, phytogenic compounds (e.g., crude extracts, phenolics, triterpenoids, polysaccharides, saponins, lectins) obtained from fruits, vegetables, and food legumes could suppress the production of inflammatory markers (e.g., C-reactive protein, IL-1, IL-6, TNF-α) or major inflammatory mediators (e.g., NO, iNOS, COX2, PGE_2_) in human intervention studies and *in vitro* cell models (212, 213). Essential oils from clove, pine, tea, garlic, cinnamon, and other compounds also possess anti-inflammatory activities that were observed *in vitro* (214, 215) and in livestock, fish, and poultry (216, 217). The anti-inflammatory mechanisms of action have not been completely understood in phytochemicals, and some research indicates that the *in vitro* anti-inflammatory or *in vivo* immune-modulatory effects are partially mediated by blocking the NF-κB activation pathway (218, 219). Other potential modes of action include inhibiting lipoxygenase and cyclooxygenase, two important enzymes in the activation of inflammatory responses (220–222).

The effects of phytochemicals on ETEC F18 and F4 infection have been evaluated in many *in vivo* pig studies. Dietary inclusion of 10 mg/kg of capsicum oleoresin, garlic botanical, or turmeric oleoresin reduced diarrhea and enhanced disease resistance of weaned pigs infected with ETEC F18 (71). Pigs fed with phytochemicals developed better intestinal health, as indicated by higher villi height, lower immune cell accumulation, and milder intestinal inflammation than infected control. The further microarray analysis confirmed that feeding these phytochemicals enhanced the integrity of membranes, especially tight junction-related genes in ileum of weaned pigs (72). In addition, the reduced intestinal inflammation by feeding phytochemicals was also observed at the transcriptional level, as indicated by the down-regulation of genes in the categories of responses to stimulus, antigen processing and presentation, and inflammatory mediators in ileal mucosa (72). Devi et al. (223) reported that supplementation of a 0.05% phytogenic combination, including clove, cinnamon, and fenugreek, improved weight gain and apparent total tract digestibility in pigs under ETEC F4 infection. Likewise, the chestnut extract containing hydrolyzable tannins was reported to reduce diarrhea and enhance the growth performance of pigs challenged with ETEC F4 (224). Cranberry supplementation in feed (10 g/kg) or *via* drinking water (1 g/L) significantly reduced the diarrhea severity of ETEC F18-infected pigs (225).

### Lysozyme

Lysozyme is a naturally existing antimicrobial enzyme that can be found in blood, liver, and many bodily secretions. It cleaves 1,4-β-linkages between N-acetylmuramic acid and N-acetyl-D-glucosamine in the peptidoglycan layer of the bacterial cell wall, thus inducing cell death (226). Lysozyme is part of innate immunity and plays an important role in limiting bacterial overgrowth at mucosal surfaces. Recent research suggests lysozyme could modulate the host immune responses to infection (227, 228). The lysozyme-mediated degradation and lysis of bacteria enhance the release of bacterial products, such as bacterial peptidoglycans, which further regulate the immune response in the host (229). However, the location of lysozyme activity, the susceptibility of bacterial peptidoglycans to lysozyme digestion, and the amount and composition of bacterial products can all modulate the degree and extent of pro-inflammatory immune responses. Thus, lysozyme could enhance or dampen the innate immune response (229). Other research also reveals that lysozyme may contribute to resolving intestinal inflammation *via* restricting bacterial growth, assisting in intestinal epithelial barrier protection, and reducing phagocyte influx and concomitant cellular inflammatory responses (227, 229, 230).

Lysozyme is one of the suitable alternatives to replace antibiotic growth promoters in swine production. Lysozyme derived from chicken eggs was reported to improve growth performance of weaned pigs, with its efficacy comparable to neomycin/oxytetracycline (101), carbadox/copper sulfate (214), or chlortetracycline/tiamulin hydrogen fumarate (231). Growing evidence also supports that the administration of lysozyme could enhance the disease resistance of weaned pigs against ETEC infection. For instance, Nyachoti et al. (114) reported that supplementation of lysozyme sourced from egg white improved intestinal development, decreased ETEC counts in the intestinal mucosa, and reduced serum pro-inflammatory cytokines in weaned pigs infected with ETEC F4. Garas et al. (232) observed that feeding lysozyme-rich goat milk reduced the incidence of diarrhea and significantly suppressed total bacteria translocation into the mesenteric lymph nodes in pigs infected with ETEC F4. Supplementing lysozyme-rich milk also reduced the relative abundance of fecal *Enterobacteriaceae* family, in which many prevalent enteric pathogens (e.g., *E. coli* and *Salmonella*) belong to. Similarly, pigs fed with human lysozyme-rich milk had a higher survival rate and reduced diarrhea when they were challenged with ETEC F4 (233). The enriched relative abundance of *Lactobacillus* in feces and enhanced intestinal integrity and mucosa immunity were also observed in these pigs (233).

## Conclusions

ETEC is one of the most predominant causes of post-weaning diarrhea in pigs. In-feed antibiotics and pharmacological ZnO were routinely added to the nursery diet to prevent diarrhea and to increase the survival rate of newly weaned pigs. However, the heavy use of medically important antimicrobials in food-producing animals induces the development and spread of antimicrobial resistance. The resistance results in the loss of effectiveness of these drugs as antimicrobial therapies, which poses a serious threat to public and animal health. The significant environmental impacts and public concerns are also highly recognized in the application of high-dose ZnO in pig feed. Thus, the exploration of alternative practices that may help reduce the reliance on antimicrobial drugs and pharmacological ZnO and address animal health needs is warranted. Accumulating evidence has confirmed the importance of nutritional interventions, including modified feeding strategies and nutrient supplements, in the control of diarrheal disease caused by ETEC. Several categories of feed additives are widely applied to nursery pigs to assist in enhancing intestinal barrier function and immunity, balancing intestinal microbiota diversity, and promoting overall health and performance. Although no single substance can fully replace the functions of in-feed antibiotics and high-dose ZnO so far, their beneficial effects on pig health and welfare are promising. Future research should focus on the development of fundamental knowledge on defining healthy gut and robust intestinal function of pigs by adopting novel approaches. Understanding the interaction of host-microbiome-nutrition is also extremely important to exploring the mechanisms of new nutritional interventions.

## Author contributions

Conceptualization: KK, YL, PJ. Writing original draft: KK. Reviewing and editing: MS, YL, and PJ. All authors contributed to the article and approved the submitted version.

## Conflict of interest

The authors declare that the research was conducted in the absence of any commercial or financial relationships that could be construed as a potential conflict of interest.

## Publisher’s note

All claims expressed in this article are solely those of the authors and do not necessarily represent those of their affiliated organizations, or those of the publisher, the editors and the reviewers. Any product that may be evaluated in this article, or claim that may be made by its manufacturer, is not guaranteed or endorsed by the publisher.

## References

[1] Lee IK Kye YC Kim G Kim HW Gu MJ Umboh J. Stress, nutrition, and intestinal immune responses in pigs — a review. Asian-Australas J Anim Sci (2016) 29:1075–82. doi: 10.5713/ajas.16.0118 PMC4932560 27189643

[2] Gimsa U Tuchscherer M Kanitz E. Psychosocial stress and immunity–what can we learn from pig studies? Front Behav Neurosci (2018) 12:64. doi: 10.3389/fnbeh.2018.00064 29666573 PMC5891618

[3] Moeser AJ Pohl CS Rajput M. Weaning stress and gastrointestinal barrier development: Implications for lifelong gut health in pigs. Anim Nutr (2017) 3:313–21. doi: 10.1016/j.aninu.2017.06.003 PMC5941262 29767141

[4] Pluske JR Miller DW Sterndale SO Turpin DL. Associations between gastrointestinal-tract function and the stress response after weaning in pigs. Anim Prod. Sci (2019) 59:2015–22. doi: 10.1071/AN19279

[5] USDA. Swine 2012; part II: Reference of swine health and health management in the united states, 2012, in: USDA–APHIS–VS–CEAH–NAHMS (2015). Available at: https://www.aphis.usda.gov/animal_health/nahms/swine/downloads/swine2012/Swine2012_dr_Trends.pdf (Accessed January 20, 2022).

[6] Amezcua R Friendship RM Dewey CE Gyles C Fairbrother JM. Presentation of postweaning *Escherichia coli* diarrhea in southern Ontario, prevalence of hemolytic e. coli serogroups involved, and their antimicrobial resistance patterns. Can J Vet Res (2002) 66:73–8. PMC226986 11989737

[7] Johnson TJ Nolan LK. Pathogenomics of the virulence plasmids of *Escherichia coli* . Microbiol Mol Biol Rev MMBR (2009) 73:750–74. doi: 10.1128/MMBR.00015-09 PMC2786578 19946140

[8] Nataro JP Kaper JB. Diarrheagenic *Escherichia coli* . Clin Microbiol Rev (1998) 11:142–201. doi: 10.1128/CMR.11.1.142 9457432 PMC121379

[9] Nagy B Fekete PZ. Enterotoxigenic *Escherichia coli* in veterinary medicine. Int J Med Microbiol (2005) 295:443–54. doi: 10.1016/j.ijmm.2005.07.003 16238018

[10] Makvana S Krilov LR. *Escherichia coli* infections. Pediatr Rev (2015) 36:167–70. doi: 10.1542/pir.36-4-167 25834220

[11] Fairbrother JM Nadeau E Gyles CL. *Escherichia coli* in postweaning diarrhea in pigs: An update on bacterial types, pathogenesis, and prevention strategies. Anim Health Res Rev (2005) 6:17–39. doi: 10.1079/ahr2005105 16164007

[12] Nagy B Fekete PZ. Enterotoxigenic *Escherichia coli* (ETEC) in farm animals. Vet Res (1999) 30:259–84. 10367358

[13] Kaper JB Nataro JP Mobley HL. Pathogenic *Escherichia coli* . Nat Rev Microbiol (2004) 2:123–40. doi: 10.1038/nrmicro818 15040260

[14] Croxen MA Law RJ Scholz R Keeney KM Wlodarska M Finlay BB. Recent advances in understanding enteric pathogenic *Escherichia coli* . Clin Microbiol Rev (2013) 26:822–80. doi: 10.1128/CMR.00022-13 PMC3811233 24092857

[15] Fleckenstein JM Munson GM Rasko DA. Enterotoxigenic *Escherichia coli* . Gut. Microbes (2013) 4:392–6. doi: 10.4161/gmic.25861 PMC3839984 23892244

[16] Mirhoseini A Amani J Nazarian S. Review on pathogenicity mechanism of enterotoxigenic *Escherichia coli* and vaccines against it. Microb Pathog (2018) 117:162–9. doi: 10.1016/j.micpath.2018.02.032 29474827

[17] Rhouma M Fairbrother JM Beaudry F Letellier A. Post weaning diarrheain pigs: risk factors and non-colistin-based control strategies. Acta Vet Scand (2017) 59:31. doi: 10.1186/s13028-017-0299-7 PMC5437690 28526080

[18] Fleckenstein JM Hardwidge PR Munson GP Rasko DA Sommerfelt H Steinsland H. Molecular mechanisms of enterotoxigenic *Escherichia coli* infection. Microbes Infect (2010) 12:89–98. doi: 10.1016/j.micinf.2009.10.002 19883790 PMC10647112

[19] Dubreuil JD Isaacson RE Schifferli DM. Animal enterotoxigenic. Escherichia coli. EcoSal. Plus (2016) 7:1–80. doi: 10.1128/ecosalplus.ESP-0006-2016 PMC5123703 27735786

[20] Frydendahl K. Prevalence of serogroups and virulence genes in *Escherichia coli* associated with postweaning diarrhoea and edema disease in pigs and a comparison of diagnostic approaches. Vet Microbiol (2002) 85:169–82. doi: 10.1016/s0378-1135(01)00504-1 11844623

[21] Thiagarajah JR Verkman A. CFTR pharmacology and its role in intestinal fluid secretion. Curr Opin Pharmacol (2003) 3:594–9. doi: 10.1016/j.coph.2003.06.012 14644010

[22] Haan L de Hirst TR. Cholera toxin: a paradigm for multi-functional engagement of cellular mechanisms. Mol Membr Biol (2004) 21:77–92. doi: 10.1080/09687680410001663267 15204437

[23] Chao AC de Sauvage FJ Dong Y Wagner J Goeddel D Gardner P. Activation of intestinal CFTR cl-channel by heat-stable enterotoxin and guanylin *via* cAMP-dependent protein kinase. EMBO J (1994) 13:1065–72. doi: 10.1002/j.1460-2075.1994.tb06355.x PMC394914 7510634

[24] Loos M Hellemans A Cox E. Optimization of a small intestinal segment perfusion model for heat-stable enterotoxin a induced secretion in pigs. Vet Immunol Immunopathol (2013) 152:82–6. doi: 10.1016/j.vetimm.2012.09.014 23159147

[25] Dreyfus LA Harville B Howard DE Shaban R Beatty DM Morris SJ. Calcium influx mediated by the *Escherichia coli* heat-stable enterotoxin b (STB). Proc Natl Acad Sci U.S.A. (1993) 90:3202–6. doi: 10.1073/pnas.90.8.3202 PMC46267 8475060

[26] Ngendahayo Mukiza C Dubreuil JD. *Escherichia coli* heat-stable toxin b impairs intestinal epithelial barrier function by altering tight junction proteins. Infect Immun (2013) 81:2819–27. doi: 10.1128/IAI.00455-13 PMC3719558 23716609

[27] Sandvig K Van Deurs B. Transport of protein toxins into cells: Pathways used by ricin, cholera toxin and shiga toxin. FEBS Lett (2002) 529:49–53. doi: 10.1016/s0014-5793(02)03182-4 12354612

[28] Sandvig K. Shiga toxins. Toxicon (2001) 39:1629–35. doi: 10.1016/s0041-0101(01)00150-7 11595626

[29] Thorpe CM Hurley BP Lincicome LL Jacewicz MS Keusch GT Acheson DW. Shiga toxins stimulate secretion of interleukin-8 from intestinal epithelial cells. Infect Immun (1999) 67:5985–93. doi: 10.1128/IAI.67.11.5985-5993.1999 PMC96984 10531258

[30] Luderitz O Freudenberg MA Galanos C Lehmann V Rietschel ET Shaw DH. Lipopolysaccharides of gram-negative bacteria. Curr Top Membr Transp. (1982) 17:79–151. doi: 10.1016/S0070-2161(08)60309-3

[31] Fenton MJ Golenbock DT. LPS-binding proteins and receptors. J Leukoc Biol (1998) 64:25–32. doi: 10.1002/jlb.64.1.25 9665271

[32] Schilling JD Mulvey MA Vincent CD Lorenz RG Hultgren SJ. Bacterial invasion augments epithelial cytokine responses to *Escherichia coli* through a lipopolysaccharide-dependent mechanism. J Immunol (2001) 166:1148–55. doi: 10.4049/jimmunol.166.2.1148 11145696

[33] Oswald IP. Role of intestinal epithelial cells in the innate immune defence of the pig intestine. Vet Res (2006) 37:359–68. doi: 10.1051/vetres:2006006 16611553

[34] McLamb BL Gibson AJ Overman EL Stahl C Moeser AJ. Early weaning stress in pigs impairs innate mucosal immune responses to enterotoxigenic e. coli challenge and exacerbates intestinal injury and clinical disease. PloS One (2013) 8:e59838. doi: 10.1371/journal.pone.0059838 23637741 PMC3634819

[35] Vermeire B Gonzalez LM Jansens RJJ Cox E Devriendt B. Porcine small intestinal organoids as a model to explore ETEC–host interactions in the gut. Vet Res (2021) 52:94. doi: 10.1186/s13567-021-00961-7 34174960 PMC8235647

[36] Johansson MEV Sjövall H Hansson GC. The gastrointestinal mucus system in health and disease. Nat Rev Gastroenterol Hepatol (2013) 10:352–61. doi: 10.1038/nrgastro.2013.35 PMC3758667 23478383

[37] Herath M Hosie S Bornstein JC Franks AE Hill-Yardin EL. The role of the gastrointestinal mucus system in intestinal homeostasis: Implications for neurological disorders. Front Cell Infect Microbiol (2020) 10:248. doi: 10.3389/fcimb.2020.00248 32547962 PMC7270209

[38] Johansson MEV Phillipson M Petersson J Velcich A Holm L Hansson GC. The inner of the two Muc2 mucin-dependent mucus layers in colon is devoid of bacteria. Proc Natl Acad Sci U.S.A. (2008) 105:15064–9. doi: 10.1073/pnas.0803124105 PMC2567493 18806221

[39] Strugnell RA Wijburg OL. The role of secretory antibodies in infection immunity. Nat Rev Microbiol (2010) 8:656–67. doi: 10.1038/nrmicro2384 20694027

[40] Kim K He Y Xiong X Ehrlich A Li X Raybould H. Dietary supplementation of *Bacillus subtilis* influenced intestinal health of weaned pigs experimentally infected with a pathogenic e. coli. J Anim Sci Biotechnol (2019) 10:1–12. doi: 10.1186/s40104-019-0364-3

[41] Xue Y Zhang H Wang H Hu J Du M Zhu M-J. Host inflammatory response inhibits *Escherichia coli* O157:H7 adhesion to gut epithelium through augmentation of mucin expression. Infect Immun (2014) 82:1921–30. doi: 10.1128/IAI.01589-13 PMC3993425 24566630

[42] Verbrugghe E Van Parys A Leyman B Boyen F Arnouts S Lundberg U. Heat-labile enterotoxin of *Escherichia coli* promotes intestinal colonization of *Salmonella enterica* . Comp Immunol Microbiol Infect Dis (2015) 43:1–7. doi: 10.1016/j.cimid.2015.09.002 26616654

[43] Kumar P Luo Q Vickers TJ Sheikh A Lewis WG Fleckenstein JM. EatA, an immunogenic protective antigen of enterotoxigenic *Escherichia coli*, degrades intestinal mucin. Infect Immun (2014) 82:500–8. doi: 10.1128/IAI.01078-13 PMC3911389 24478066

[44] Luo Q Kumar P Vickers TJ Sheikh A Lewis WG Rasko DA. Enterotoxigenic *Escherichia coli* secretes a highly conserved mucin-degrading metalloprotease to effectively engage intestinal epithelial cells. Infect Immun (2013) 82:509–21. doi: 10.1128/IAI.01106-13 PMC3911403 24478067

[45] Sheikh A Wangdi T Vickers TJ Aaron B Palmer M Miller MJ. Enterotoxigenic *Escherichia coli* degrades the host MUC2 mucin barrier to facilitate critical pathogen-enterocyte interactions in human small intestine. Infect Immun (2022) 90:e0057221. doi: 10.1128/IAI.00572-21 PMC8853678 34807735

[46] Tsukita S Furuse M Itoh M. Multifunctional strands in tight junctions. Nat Rev Mol Cell Biol (2001) 2:285–93. doi: 10.1038/35067088 11283726

[47] Baumgart DC Dignass AU. Intestinal barrier function. Curr Opin Clin Nutr Metab Care (2002) 5:685–94. doi: 10.1097/00075197-200211000-00012 12394645

[48] González-Mariscal L Betanzos A Nava P Jaramillo BE. Tight junction proteins. Prog Biophys Mol Biol (2003) 81:1–44. doi: 10.1016/S0079-6107(02)00037-8 12475568

[49] Bauer H Zweimueller-Mayer J Steinbacher P Lametschwandtner A Bauer HC. The dual role of zonula occludens (ZO) proteins. J BioMed Biotechnol (2010) 2010:e402593. doi: 10.1155/2010/402593 PMC2836178 20224657

[50] Otani T Furuse M. Tight junction structure and function revisited. Trends Cell Biol (2020) 30:805–17. doi: 10.1016/j.tcb.2020.08.004 32891490

[51] Zihni C Mills C Matter K Balda MS. Tight junctions: From simple barriers to multifunctional molecular gates. Nat Rev Mol Cell Biol (2016) 17:564–80. doi: 10.1038/nrm.2016.80 27353478

[52] Berkes J Viswanathan VK Savkovic SD Hecht G. Intestinal epithelial responses to enteric pathogens: Effects on the tight junction barrier, ion transport, and inflammation. Gut (2003) 52:439–51. doi: 10.1136/gut.52.3.439 PMC1773546 12584232

[53] Ewaschuk JB Murdoch GK Johnson IR Madsen KL Field CJ. Glutamine supplementation improves intestinal barrier function in a weaned piglet model of *Escherichia coli* infection. Br J Nutr (2011) 106:870–7. doi: 10.1017/S0007114511001152 21736826

[54] Yu Q Wang Z Yang Q. *Lactobacillus amylophilus* D14 protects tight junction from enteropathogenic bacteria damage in caco-2 cells. J Dairy Sci (2012) 95:5580–7. doi: 10.3168/jds.2012-5540 22884350

[55] Gao Y Han F Huang X Rong Y Yi H Wang Y. Changes in gut microbial populations, intestinal morphology, expression of tight junction proteins, and cytokine production between two pig breeds after challenge with *Escherichia coli* K88: A comparative study. J Anim Sci (2013) 91:5614–25. doi: 10.2527/jas.2013-6528 24126267

[56] Yang KM Jiang ZY Zheng CT Wang L Yang XF. Effect of *Lactobacillus plantarum* on diarrhea and intestinal barrier function of young piglets challenged with enterotoxigenic *Escherichia coli* K88. J Anim Sci (2014) 92:1496–503. doi: 10.2527/jas.2013-6619 24492550

[57] Wu Y Zhu C Chen Z Chen Z Zhang W Ma X. Protective effects of *Lactobacillus plantarum* on epithelial barrier disruption caused by enterotoxigenic *Escherichia coli* in intestinal porcine epithelial cells. Vet Immunol Immunopathol (2016) 172:55–63. doi: 10.1016/j.vetimm.2016.03.005 27032504

[58] Yang G-Y Zhu Y-H Zhang W Zhou D Zhai C-C Wang J-F. Influence of orally fed a select mixture of *Bacillus* probiotics on intestinal T-cell migration in weaned MUC4 resistant pigs following *Escherichia coli* challenge. Vet Res (2016) 47:71. doi: 10.1186/s13567-016-0355-8 27424033 PMC4947265

[59] Li H-H Li Y-P Zhu Q Qiao J-Y Wang W-J. Dietary supplementation with *Clostridium butyricum* helps to improve the intestinal barrier function of weaned piglets challenged with enterotoxigenic *Escherichia coli* K88. J Appl Microbiol (2018) 125:964–75. doi: 10.1111/jam.13936 29851202

[60] Li Q Burrough ER Gabler NK Loving CL Sahin O Gould SA. A soluble and highly fermentable dietary fiber with carbohydrases improved gut barrier integrity markers and growth performance in F18 ETEC challenged pigs. J Anim Sci (2019) 97:2139–53. doi: 10.1093/jas/skz093 PMC6488326 30888017

[61] Becker SL Li Q Burrough ER Kenne D Sahin O Gould SA. Effects of an F18 enterotoxigenic escherichia coli challenge on growth performance, immunological status, and gastrointestinal structure of weaned pigs and the potential protective effect of direct-fed microbial blends. J Anim Sci (2020) 98:skaa113. doi: 10.1093/jas/skaa113 32300795 PMC7228676

[62] Hu C Song J Li Y Luan Z Zhu K. Diosmectite–zinc oxide composite improves intestinal barrier function, modulates expression of pro-inflammatory cytokines and tight junction protein in early weaned pigs. Br J Nutr (2013) 110:681–8. doi: 10.1017/S0007114512005508 23308387

[63] Berg RD. Bacterial translocation from the gastrointestinal tract. Trends Microbiol (1995) 3:149–54. doi: 10.1016/s0966-842x(00)88906-4 7613757

[64] Nagpal R Yadav H. Bacterial translocation from the gut to the distant organs: An overview. Ann Nutr Metab (2017) 71 Suppl 1:11–6. doi: 10.1159/000479918 28950279

[65] Balzan S De Almeida Quadros C De Cleva R Zilberstein B Cecconello I. Bacterial translocation: Overview of mechanisms and clinical impact. J Gastroenterol Hepatol (2007) 22:464–71. doi: 10.1111/j.1440-1746.2007.04933.x 17376034

[66] Almeida JAS Liu Y Song M Lee JJ Gaskins HR Maddox CW. *Escherichia coli* challenge and one type of smectite alter intestinal barrier of pigs. J Anim Sci Biotechnol (2013) 4:52. doi: 10.1186/2049-1891-4-52 24359581 PMC3897994

[67] Holmgren J Czerkinsky C. Mucosal immunity and vaccines. Nat Med (2005) 11:S45–53. doi: 10.1038/nm1213 15812489

[68] Fournier B Parkos C. The role of neutrophils during intestinal inflammation. Mucosal Immunol (2012) 5:354–66. doi: 10.1038/mi.2012.24 22491176

[69] Kolaczkowska E Kubes P. Neutrophil recruitment and function in health and inflammation. Nat Rev Immunol (2013) 13:159–75. doi: 10.1038/nri3399 23435331

[70] Sugimoto MA Sousa LP Pinho V Perretti M Teixeira MM. Resolution of inflammation: What controls its onset? Front Immunol (2016) 7:160. doi: 10.3389/fimmu.2016.00160 27199985 PMC4845539

[71] Liu Y Song M Che TM Almeida J a S Lee JJ Bravo D. Dietary plant extracts alleviate diarrhea and alter immune responses of weaned pigs experimentally infected with a pathogenic. Escherichia coli. J Anim Sci (2013) 91:5294–306. doi: 10.2527/jas.2012-6194 24045466

[72] Liu Y Song M Che TM Lee JJ Bravo D Maddox CW. Dietary plant extracts modulate gene expression profiles in ileal mucosa of weaned pigs after an *Escherichia coli* infection. J Anim Sci (2014) 92:2050–62. doi: 10.2527/jas.2013-6422 24663182

[73] Zhou X Giron JA Torres AG Crawford JA Negrete E Vogel SN. Flagellin of enteropathogenic *Escherichia coli* stimulates interleukin-8 production in T84 cells. Infect Immun (2003) 71:2120–9. doi: 10.1128/IAI.71.4.2120-2129.2003 PMC152053 12654834

[74] Savkovic SD Koutsouris A Hecht G. Activation of NF-kappaB in intestinal epithelial cells by enteropathogenic escherichia coli. Am J Physiol (1997) 273:C1160–1167. doi: 10.1152/ajpcell.1997.273.4.C1160 9357759

[75] Savkovic SD Ramaswamy A Koutsouris A Hecht G. EPEC-activated ERK1/2 participate in inflammatory response but not tight junction barrier disruption. Am J Physiol Gastrointest Liver Physiol (2001) 281:G890–898. doi: 10.1152/ajpgi.2001.281.4.G890 11557508

[76] Ceponis PJM McKay DM Ching JCY Pereira P Sherman PM. Enterohemorrhagic *Escherichia coli* O157:H7 disrupts Stat1-mediated gamma interferon signal transduction in epithelial cells. Infect Immun (2003) 71:1396–404. doi: 10.1128/IAI.71.3.1396-1404.2003 PMC148815 12595457

[77] Chen G Shaw MH Kim Y-G Nunez G. NOD-like receptors: role in innate immunity and inflammatory disease. Annu Rev Pathol (2009) 4:365–98. doi: 10.1146/annurev.pathol.4.110807.092239 18928408

[78] Bone RC. Gram-negative sepsis: Background, clinical features, and intervention. Chest (1991) 100:802–8. doi: 10.1378/chest.100.3.802 1889276

[79] Jesmok G Lindsey C Duerr M Fournel M Emerson T. Efficacy of monoclonal antibody against human recombinant tumor necrosis factor in e. coli-challenged swine. Am J Pathol (1992) 141:1197–207. PMC1886673 1443053

[80] Song M Liu Y Soares JA Che TM Osuna O Maddox CW. Dietary clays alleviate diarrhea of weaned pigs. J Anim Sci (2012) 90:345–60. doi: 10.2527/jas.2010-3662 21908641

[81] Kim K Ehrlich A Perng V Chase JA Raybould H Li X. Algae-derived β-glucan enhanced gut health and immune responses of weaned pigs experimentally infected with a pathogenic E. coli. Anim Feed Sci Technol (2019) 248:114–25. doi: 10.1016/j.anifeedsci.2018.12.004

[82] Xu C Wang Y Sun R Qiao X Shang X Niu W. Modulatory effects of vasoactive intestinal peptide on intestinal mucosal immunity and microbial community of weaned piglets challenged by an enterotoxigenic *Escherichia coli* (K88). PloS One (2014) 9:e104183. doi: 10.1371/journal.pone.0104183 25101851 PMC4125177

[83] Lee CY Kim SJ Park BC Han JH. Effects of dietary supplementation of bacteriophages against enterotoxigenic *Escherichia coli* (ETEC) K88 on clinical symptoms of post-weaning pigs challenged with the ETEC pathogen. J Anim Physiol Anim Nutr (2017) 101:88–95. doi: 10.1111/jpn.12513 27271838

[84] Li H Liu X Shang Z Qiao J. *Clostridium butyricum* helps to alleviate inflammation in weaned piglets challenged with enterotoxigenic *Escherichia coli* K88. Front Vet Sci (2021) 8:683863. doi: 10.3389/fvets.2021.683863 34277756 PMC8282889

[85] Kamada N Seo S-U Chen GY Núñez G. Role of the gut microbiota in immunity and inflammatory disease. Nat Rev Immunol (2013) 13:321–35. doi: 10.1038/nri3430 23618829

[86] Gresse R Chaucheyras-Durand F Fleury MA Van de Wiele T Forano E Blanquet-Diot S. Gut microbiota dysbiosis in postweaning piglets: Understanding the keys to health. Trends Microbiol (2017) 25:851–73. doi: 10.1016/j.tim.2017.05.004 28602521

[87] Isaacson R Kim HB. The intestinal microbiome of the pig. Anim Health Res Rev (2012) 13:100–9. doi: 10.1017/S1466252312000084 22853934

[88] Fouhse JM Zijlstra RT Willing BP. The role of gut microbiota in the health and disease of pigs. Anim Front (2016) 6:30–6. doi: 10.2527/af.2016-0031

[89] Dou S Gadonna-Widehem P Rome V Hamoudi D Rhazi L Lakhal L. Characterisation of early-life fecal microbiota in susceptible and healthy pigs to post-weaning diarrhoea. PLoS One (2017) 12:e0169851. doi: 10.1371/journal.pone.0169851 28072880 PMC5225014

[90] Wang X Tsai T Deng F Wei X Chai J Knapp J. Longitudinal investigation of the swine gut microbiome from birth to market reveals stage and growth performance associated bacteria. Microbiome (2019) 7:109. doi: 10.1186/s40168-019-0721-7 31362781 PMC6664762

[91] Bin P Tang Z Liu S Chen S Xia Y Liu J. Intestinal microbiota mediates enterotoxigenic *Escherichia coli*-induced diarrhea in piglets. BMC Vet Res (2018) 14:385. doi: 10.1186/s12917-018-1704-9 30518356 PMC6282381

[92] Rinninella E Raoul P Cintoni M Franceschi F Miggiano GAD Gasbarrini A. What is the healthy gut microbiota composition? a changing ecosystem across age, environment, diet, and diseases. Microorganisms (2019) 7:14. doi: 10.3390/microorganisms7010014 PMC6351938 30634578

[93] Costa MO Chaban B Harding JCS Hill JE. Characterization of the fecal microbiota of pigs before and after inoculation with “*Brachyspira hampsonii*”. PloS One (2014) 9:e106399. doi: 10.1371/journal.pone.0106399 25166307 PMC4148400

[94] Song D Peng Q Chen Y Zhou X Zhang F Li A. Altered gut microbiota profiles in sows and neonatal piglets associated with porcine epidemic diarrhea virus infection. Sci Rep (2017) 7:17439. doi: 10.1038/s41598-017-17830-z 29234140 PMC5727058

[95] Pollock J Gally DL Glendinning L Tiwari R Hutchings MR Houdijk JGM. Analysis of temporal fecal microbiota dynamics in weaner pigs with and without exposure to enterotoxigenic *Escherichia coli* . J Anim Sci (2018) 96:3777–90. doi: 10.1093/jas/sky260 PMC6127793 29982429

[96] Duarte ME Tyus J Kim SW. Synbiotic effects of enzyme and probiotics on intestinal health and growth of newly weaned pigs challenged with enterotoxigenic F18+*Escherichia coli* . Front Vet Sci (2020) 7:573. doi: 10.3389/fvets.2020.00573 33033721 PMC7509054

[97] Li Q Peng X Burrough ER Sahin O Gould SA Gabler NK. Dietary soluble and insoluble fiber with or without enzymes altered the intestinal microbiota in weaned pigs challenged with enterotoxigenic e. coli F18. Front Microbiol (2020) 11:1110. doi: 10.3389/fmicb.2020.01110 32536908 PMC7267687

[98] Wang W Wang Y Hao X Duan Y Meng Z An X. Dietary fermented soybean meal replacement alleviates diarrhea in weaned piglets challenged with enterotoxigenic *Escherichia coli* K88 by modulating inflammatory cytokine levels and cecal microbiota composition. BMC Vet Res (2020) 16:245. doi: 10.1186/s12917-020-02466-5 32664940 PMC7362456

[99] Yue Y He Z Zhou Y Paul Ross R Stanton C Zhao J. *Lactobacillus plantarum* relieves diarrhea caused by enterotoxin-producing *Escherichia coli* through inflammation modulation and gut microbiota regulation. Food Funct (2020) 11:10362–74. doi: 10.1039/D0FO02670K 33220669

[100] Wells JE Oliver WT Yen JT. The effects of dietary additives on faecal levels of *Lactobacillus* spp., coliforms, and *Escherichia coli*, and faecal prevalence of *Salmonella* spp. and *Campylobacter* spp. in US production nursery swine. J Appl Microbiol (2010) 108:306–14. doi: 10.1111/j.1365-2672.2009.04423.x 19614855

[101] May KD Wells JE Maxwell CV Oliver WT. Granulated lysozyme as an alternative to antibiotics improves growth performance and small intestinal morphology of 10-day-old pigs. J Anim Sci (2012) 90:1118–25. doi: 10.2527/jas.2011-4297 22064735

[102] Macheboeuf P Contreras-Martel C Job V Dideberg O Dessen A. Penicillin binding proteins: Key players in bacterial cell cycle and drug resistance processes. FEMS Microbiol Rev (2006) 30:673–91. doi: 10.1111/j.1574-6976.2006.00024.x 16911039

[103] Cho H Uehara T Bernhardt TG. Beta-lactam antibiotics induce a lethal malfunctioning of the bacterial cell wall synthesis machinery. Cell (2014) 159:1300–11. doi: 10.1016/j.cell.2014.11.017 PMC4258230 25480295

[104] Poehlsgaard J Douthwaite S. The bacterial ribosome as a target for antibiotics. Nat Rev Microbiol (2005) 3:870–81. doi: 10.1038/nrmicro1265 16261170

[105] Wilson DN. Ribosome-targeting antibiotics and mechanisms of bacterial resistance. Nat Rev Microbiol (2014) 12:35–48. doi: 10.1038/nrmicro3155 24336183

[106] Bulkley D Innis CA Blaha G Steitz TA. Revisiting the structures of several antibiotics bound to the bacterial ribosome. Proc Natl Acad Sci U.S.A. (2010) 107:17158–63. doi: 10.1073/pnas.1008685107 PMC2951403 20876130

[107] Pham TDM Ziora ZM Blaskovich MAT. Quinolone antibiotics. MedChemComm (2019) 10:1719–39. doi: 10.1039/C9MD00120D PMC6836748 31803393

[108] Bush NG Diez-Santos I Abbott LR Maxwell A. Quinolones: Mechanism, lethality and their contributions to antibiotic resistance. Molecules (2020) 25:5662. doi: 10.3390/molecules25235662 PMC7730664 33271787

[109] Field MS Kamynina E Chon J Stover PJ. Nuclear folate metabolism. Annu Rev Nutr (2018) 38:219–43. doi: 10.1146/annurev-nutr-071714-034441 PMC11788913 30130467

[110] Fernandez-Villa D Aguilar MR Rojo L. Folic acid antagonists: Antimicrobial and immunomodulating mechanisms and applications. Int J Mol Sci (2019) 20:4996. doi: 10.3390/ijms20204996 PMC6829374 31601031

[111] Capasso C Supuran CT. Sulfa and trimethoprim-like drugs–antimetabolites acting as carbonic anhydrase, dihydropteroate synthase and dihydrofolate reductase inhibitors. J Enzyme Inhib. Med Chem (2014) 29:379–87. doi: 10.3109/14756366.2013.787422 23627736

[112] Cromwell GL. Why and how antibiotics are used in swine production. Anim Biotechnol (2002) 13:7–27. doi: 10.1081/ABIO-120005767 12212945

[113] Li J. Current status and prospects for in-feed antibiotics in the different stages of pork production–a review. Asian-Australas J Anim Sci (2017) 30:1667–73. doi: 10.5713/ajas.17.0418 PMC5666167 28823126

[114] Nyachoti CM Kiarie E Bhandari SK Zhang G Krause DO. Weaned pig responses to *Escherichia coli* K88 oral challenge when receiving a lysozyme supplement. J Anim Sci (2012) 90:252–60. doi: 10.2527/jas.2010-3596 21890507

[115] Pan L Zhao PF Ma XK Shang QH Xu YT Long SF. Probiotic supplementation protects weaned pigs against enterotoxigenic *Escherichia coli* K88 challenge and improves performance similar to antibiotics. J Anim Sci (2017) 95:2627–39. doi: 10.2527/jas.2016.1243 28727032

[116] Lee JS Awji EG Lee SJ Tassew DD Park YB Park KS. Effect of *Lactobacillus plantarum* CJLP243 on the growth performance and cytokine response of weaning pigs challenged with enterotoxigenic *Escherichia coli* . J Anim Sci (2012) 90:3709–17. doi: 10.2527/jas.2011-4434 22859771

[117] Trevisi P Colombo M Priori D Fontanesi L Galimberti G Calò G. Comparison of three patterns of feed supplementation with live *Saccharomyces cerevisiae* yeast on postweaning diarrhea, health status, and blood metabolic profile of susceptible weaning pigs orally challenged with *Escherichia coli* F4ac. J Anim Sci (2015) 93:2225–33. doi: 10.2527/jas.2014-8539 26020319

[118] Kim SJ Kwon CH Park BC Lee CY Han JH. Effects of a lipid-encapsulated zinc oxide dietary supplement, on growth parameters and intestinal morphology in weanling pigs artificially infected with enterotoxigenic *Escherichia coli* . J Anim Sci Technol (2015) 57:4. doi: 10.1186/s40781-014-0038-9 26290724 PMC4540299

[119] Owusu-Asiedu A Nyachoti CM Marquardt RR. Response of early-weaned pigs to an enterotoxigenic *Escherichia coli* (K88) challenge when fed diets containing spray-dried porcine plasma or pea protein isolate plus egg yolk antibody, zinc oxide, fumaric acid, or antibiotic1. J Anim Sci (2003) 81:1790–8. doi: 10.2527/2003.8171790x 12854816

[120] Hong J Ariyibi S Antony L Scaria J Dilberger-Lawson S Francis D. Growth performance and gut health of *Escherichia coli*–challenged weaned pigs fed canola meal-containing diet. J Anim Sci (2021) 99:skab196. doi: 10.1093/jas/skab196 34159354 PMC8349558

[121] He Y Kim K Kovanda L Jinno C Song M Chase J. *Bacillus subtilis*: A potential growth promoter in weaned pigs in comparison to carbadox. J Anim Sci (2020) 98:skaa290. doi: 10.1093/jas/skaa290 32877510 PMC7523599

[122] Kim K He Y Jinno C Kovanda L Li X Song M. Trace amounts of antibiotic exacerbated diarrhea and systemic inflammation of weaned pigs infected with a pathogenic escherichia coli. J Anim Sci (2021) 99:skab073. doi: 10.1093/jas/skab073 33693730 PMC8480179

[123] Kim K He Y Jinno C Kovanda L Li X Bravo D. Supplementation of oligosaccharide-based polymer enhanced growth and disease resistance of weaned pigs by modulating intestinal integrity and systemic immunity. J Anim Sci Biotechnol (2022) 13:10. doi: 10.1186/s40104-021-00655-2 35016715 PMC8753815

[124] Ben Y Fu C Hu M Liu L Wong MH Zheng C. Human health risk assessment of antibiotic resistance associated with antibiotic residues in the environment: A review. Environ Res (2019) 169:483–93. doi: 10.1016/j.envres.2018.11.040 30530088

[125] Pettigrew JE. Reduced use of antibiotic growth promoters in diets fed to weanling pigs: Dietary tools, part 1. Anim Biotechnol (2006) 17:207–15. doi: 10.1080/10495390600956946 17127531

[126] Lalles J-P Bosi P Smidt H Stokes CR. Nutritional management of gut health in pigs around weaning. Proc Nutr Soc (2007) 66:260–8. doi: 10.1017/S0029665107005484 17466106

[127] Heo JM Opapeju FO Pluske JR Kim JC Hampson DJ Nyachoti CM. Gastrointestinal health and function in weaned pigs: A review of feeding strategies to control post-weaning diarrhoea without using in-feed antimicrobial compounds. J Anim Physiol Anim Nutr (2013) 97:207–37. doi: 10.1111/j.1439-0396.2012.01284.x 22416941

[128] Liu Y Espinosa CD Abelilla JJ Casas GA Lagos LV Lee SA. Non-antibiotic feed additives in diets for pigs: A review. Anim Nutr (2018) 4:113–25. doi: 10.1016/j.aninu.2018.01.007 PMC6103469 30140751

[129] Suttle NF. Mineral nutrition of livestock. 4th ed. Oxfordshire, OX, UK: CABI (2010).

[130] Haase H Rink L. Zinc signals and immune function. BioFactors (2014) 40:27–40. doi: 10.1002/biof.1114 23804522

[131] Maywald M Wessels I Rink L. Zinc signals and immunity. Int J Mol Sci (2017) 18:2222. doi: 10.3390/ijms18102222 PMC5666901 29064429

[132] Wessels I Maywald M Rink L. Zinc as a gatekeeper of immune function. Nutrients (2017) 9:1286. doi: 10.3390/nu9121286 PMC5748737 29186856

[133] Bonaventura P Benedetti G Albarède F Miossec P. Zinc and its role in immunity and inflammation. Autoimmun Rev (2015) 14:277–85. doi: 10.1016/j.autrev.2014.11.008 25462582

[134] Poulsen HD. Zinc oxide for weanling piglets. Acta Agric Scand Sect. — Anim Sci (1995) 45:159–67. doi: 10.1080/09064709509415847

[135] Hill GM Cromwell GL Crenshaw TD Dove CR Ewan RC Knabe DA. Growth promotion effects and plasma changes from feeding high dietary concentrations of zinc and copper to weanling pigs (regional study). J Anim Sci (2000) 78:1010–6. doi: 10.2527/2000.7841010x 10784192

[136] Espinosa CD Stein HH. Digestibility and metabolism of copper in diets for pigs and influence of dietary copper on growth performance, intestinal health, and overall immune status: A review. J Anim Sci Biotechnol (2021) 12:13. doi: 10.1186/s40104-020-00533-3 33431053 PMC7798237

[137] Pearce SC Sanz Fernandez M-V Torrison J Wilson ME Baumgard LH Gabler NK. Dietary organic zinc attenuates heat stress–induced changes in pig intestinal integrity and metabolism. J Anim Sci (2015) 93:4702–13. doi: 10.2527/jas.2015-9018 26523563

[138] Hu C Song J You Z Luan Z Li W. Zinc oxide–montmorillonite hybrid influences diarrhea, intestinal mucosal integrity, and digestive enzyme activity in weaned pigs. Biol Trace Elem. Res (2012) 149:190–6. doi: 10.1007/s12011-012-9422-9 22539019

[139] Zhang B Guo Y. Supplemental zinc reduced intestinal permeability by enhancing occludin and zonula occludens protein-1 (ZO-1) expression in weaning piglets. Br J Nutr (2009) 102:687–93. doi: 10.1017/S0007114509289033 19267955

[140] Shen J Chen Y Wang Z Zhou A He M Mao L. Coated zinc oxide improves intestinal immunity function and regulates microbiota composition in weaned piglets. Br J Nutr (2014) 111:2123–34. doi: 10.1017/S0007114514000300 24606984

[141] Roselli M Finamore A Garaguso I Britti MS Mengheri E. Zinc oxide protects cultured enterocytes from the damage induced by *Escherichia coli* . J Nutr (2003) 133:4077–82. doi: 10.1093/jn/133.12.4077 14652351

[142] Katouli M Melin L Jensen-Waern M Wallgren P Mollby R. The effect of zinc oxide supplementation on the stability of the intestinal flora with special reference to composition of coliforms in weaned pigs. J Appl Microbiol (1999) 87:564–73. doi: 10.1046/j.1365-2672.1999.00853.x 10583685

[143] Burrough ER De Mille C Gabler NK. Zinc overload in weaned pigs: Tissue accumulation, pathology, and growth impacts. J Vet Diagn Invest (2019) 31:537–45. doi: 10.1177/1040638719852144 PMC6857036 31170897

[144] Komatsu T Sugie K Inukai N Eguchi O Oyamada T Sawada H. Chronic pancreatitis in farmed pigs fed excessive zinc oxide. J Vet Diagn Invest (2020) 32:689–94. doi: 10.1177/1040638720944368 PMC7488960 32715990

[145] Bednorz C Oelgeschläger K Kinnemann B Hartmann S Neumann K Pieper R. The broader context of antibiotic resistance: Zinc feed supplementation of piglets increases the proportion of multi-resistant *Escherichia coli in vivo* . Int J Med Microbiol (2013) 303:396–403. doi: 10.1016/j.ijmm.2013.06.004 23856339

[146] Yazdankhah S Rudi K Bernhoft A. Zinc and copper in animal feed–development of resistance and co-resistance to antimicrobial agents in bacteria of animal origin. Microb Ecol Health Dis (2014) 25 :25862–8. doi: 10.3402/mehd.v25.25862 PMC4179321 25317117

[147] Vahjen W Pietruszyńska D Starke IC Zentek J. High dietary zinc supplementation increases the occurrence of tetracycline and sulfonamide resistance genes in the intestine of weaned pigs. Gut. Pathog (2015) 7:23. doi: 10.1186/s13099-015-0071-3 26322131 PMC4551370

[148] World Health Organization (WHO). Health and nutritional properties of probiotics in food including powder milk with live lactic acid bacteria. Cordoba, Argentina: Report from FAO/WHO Expert Consultation (2001) p. 1–4.

[149] Stein HH Kil DY. Reduced use of antibiotic growth promoters in diets fed to weanling pigs: Dietary tools, part 2. Anim Biotechnol (2006) 17:217–31. doi: 10.1080/10495390600957191 17127532

[150] Le Bon M Davies HE Glynn C Thompson C Madden M Wiseman J. Influence of probiotics on gut health in the weaned pig. Livest Sci (2010) 133:179–81. doi: 10.1016/j.livsci.2010.06.058

[151] Liao SF Nyachoti M. Using probiotics to improve swine gut health and nutrient utilization. Anim Nutr (2017) 3:331–43. doi: 10.1016/j.aninu.2017.06.007 PMC5941265 29767089

[152] Cutting SM. *Bacillus* probiotics. Food Microbiol (2011) 28:214–20. doi: 10.1016/j.fm.2010.03.007 21315976

[153] Celandroni F Vecchione A Cara A Mazzantini D Lupetti A Ghelardi E. Identification of *Bacillus* species: Implication on the quality of probiotic formulations. PloS One (2019) 14:e0217021. doi: 10.1371/journal.pone.0217021 31107885 PMC6527297

[154] Sansinenea E. Bacillus spp.: As plant growth-promoting bacteria Vol. . p. . Singapore: Springer (2019) p. 225–37. doi: 10.1007/978-981-13-5862-3_11

[155] Hoa NT Baccigalupi L Huxham A Smertenko A Van PH Ammendola S. Characterization of *Bacillus* species used for oral bacteriotherapy and bacterioprophylaxis of gastrointestinal disorders. Appl Environ Microbiol (2000) 66:5241–7. doi: 10.1128/AEM.66.12.5241-5247.2000 PMC92451 11097897

[156] Barbosa Teresa M Serra Cláudia R La Ragione Roberto M Woodward Martin J Henriques Adriano O. Screening for *Bacillus* isolates in the broiler gastrointestinal tract. Appl Environ Microbiol (2005) 71:968–78. doi: 10.1128/AEM.71.2.968-978.2005 PMC546680 15691955

[157] Larsen N Thorsen L Kpikpi EN Stuer-Lauridsen B Cantor MD Nielsen B. Characterization of *Bacillus* spp. strains for use as probiotic additives in pig feed. Appl Microbiol Biotechnol (2014) 98:1105–18. doi: 10.1007/s00253-013-5343-6 24201893

[158] Mingmongkolchai S Panbangred W. *Bacillus* probiotics: an alternative to antibiotics for livestock production. J Appl Microbiol (2018) 124:1334–46. doi: 10.1111/jam.13690 29316021

[159] Lewton JR Woodward AD Moser RL Thelen KM Moeser AJ Trottier NL. Effects of a multi-strain *Bacillus subtilis*-based direct-fed microbial on weanling pig growth performance and nutrient digestibility. Transl Anim Sci (2021) 5:txab058. doi: 10.1093/tas/txab058 34278233 PMC8281103

[160] Guo M Wu F Hao G Qi Q Li R Li N. *Bacillus subtilis* improves immunity and disease resistance in rabbits. Front Immunol (2017) 8:354. doi: 10.3389/fimmu.2017.00354 28424690 PMC5372816

[161] Rhayat L Maresca M Nicoletti C Perrier J Brinch KS Christian S. Effect of *Bacillus subtilis* strains on intestinal barrier function and inflammatory response. Front Immunol (2019) 10:564. doi: 10.3389/fimmu.2019.00564 30984172 PMC6449611

[162] Zhang W Zhu Y-H Zhou D Wu Q Song D Dicksved J. Oral administration of a select mixture of *Bacillus* probiotics affects the gut microbiota and goblet cell function following *Escherichia coli* challenge in newly weaned pigs of genotype MUC4 that are supposed to be enterotoxigenic e. coli F4ab/ac receptor negative. Appl Environ Microbiol (2017) 83:e02747–16. doi: 10.1128/AEM.02747-16 PMC5244294 27881419

[163] Luise D Bertocchi M Motta V Salvarani C Bosi P Luppi A. *Bacillus* sp. probiotic supplementation diminish the *Escherichia coli* F4ac infection in susceptible weaned pigs by influencing the intestinal immune response, intestinal microbiota and blood metabolomics. J Anim Sci Biotechnol (2019) 10:74. doi: 10.1186/s40104-019-0380-3 31528339 PMC6740008

[164] Helander IM von Wright A Mattila-Sandholm T-M. Potential of lactic acid bacteria and novel antimicrobials against gram-negative bacteria. Trends Food Sci Technol (1997) 8:146–50. doi: 10.1016/S0924-2244(97)01030-3

[165] Brashears MM Amezquita A Jaroni D. Lactic acid bacteria and their uses in animal feeding to improve food safety. Adv Food Nutr Res (2005) 50:1–31. doi: 10.1016/S1043-4526(05)50001-9 16263426

[166] Malago JJ Koninkx JFJG. Probiotic-pathogen interactions and entericcytoprotection. Dordrecht: Springer Netherlands (2011) p. 289–311. doi: 10.1007/978-94-007-0386-5_13

[167] Li X-Q Zhu Y-H Zhang H-F Yue Y Cai Z-X Lu Q-P. Risks associated with high-dose lactobacillus rhamnosus in an *Escherichia coli* model of piglet diarrhoea: Intestinal microbiota and immune imbalances. PLoS One (2012) 7:e40666. doi: 10.1371/journal.pone.0040666 22848393 PMC3407149

[168] Perdigon G Maldonado Galdeano C Valdez JC Medici M. Interaction of lactic acid bacteria with the gut immune system. Eur J Clin Nutr (2002) 56:S21–6. doi: 10.1038/sj.ejcn.1601658 12556943

[169] Tsai Y-T Cheng P-C Pan T-M. The immunomodulatory effects of lactic acid bacteria for improving immune functions and benefits. Appl Microbiol Biotechnol (2012) 96:853–62. doi: 10.1007/s00253-012-4407-3 23001058

[170] Mack DR Michail S Wei S McDougall L Hollingsworth MA. Probiotics inhibit enteropathogenic e. coli adherence *in vitro* by inducing intestinal mucin gene expression. Am J Physiol-Gastrointest Liver Physiol (1999) 276:G941–50. doi: 10.1152/ajpgi.1999.276.4.G941 10198338

[171] Yang F Hou C Zeng X Qiao S. The use of lactic acid bacteria as a probiotic in swine diets. Pathogens (2015) 4:34–45. doi: 10.3390/pathogens4010034 25633489 PMC4384071

[172] Kogan G Kocher A. Role of yeast cell wall polysaccharides in pig nutrition and health protection. Livest Sci (2007) 109:161–5. doi: 10.1016/j.livsci.2007.01.134

[173] Liu Y Wu Q Wu X Algharib SA Gong F Hu J. Structure, preparation, modification, and bioactivities of β-glucan and mannan from yeast cell wall: A review. Int J Biol Macromol (2021) 173:445–56. doi: 10.1016/j.ijbiomac.2021.01.125 33497691

[174] Vannucci L Krizan J Sima P Stakheev D Caja F Rajsiglova L. Immunostimulatory properties and antitumor activities of glucans (Review). Int J Oncol (2013) 43:357–64. doi: 10.3892/ijo.2013.1974 PMC3775562 23739801

[175] Ferreira IMPLVO Pinho O Vieira E Tavarela JG. Brewer’s *Saccharomyces* yeast biomass: Characteristics and potential applications. Trends Food Sci Technol (2010) 21:77–84. doi: 10.1016/j.tifs.2009.10.008

[176] Broadway PR Carroll JA Sanchez NCB. Live yeast and yeast cell wall supplements enhance immune function and performance in food-producing livestock: A review. Microorganisms (2015) 3:417–27. doi: 10.3390/microorganisms3030417 PMC5023245 27682097

[177] Trevisi P Latorre R Priori D Luise D Archetti I Mazzoni M. Effect of feed supplementation with live yeast on the intestinal transcriptome profile of weaning pigs orally challenged with *Escherichia coli* F4. Animal (2017) 11:33–44. doi: 10.1017/S1751731116001178 27358089

[178] Gibson GR Roberfroid MB. Dietary modulation of the human colonic microbiota: Introducing the concept of prebiotics. J Nutr (1995) 125:1401–12. doi: 10.1093/jn/125.6.1401 7782892

[179] Gibson GR Probert HM Loo JV Rastall RA Roberfroid MB. Dietary modulation of the human colonic microbiota: Updating the concept of prebiotics. Nutr Res Rev (2004) 17:259–75. doi: 10.1079/NRR200479 19079930

[180] Zimmermann B Bauer E Mosenthin R. Pro- and prebiotics in pig nutrition-potential modulators of gut health? J Anim Feed Sci (2001) 10:47–56. doi: 10.22358/jafs/67940/2001

[181] Gibson GR McCartney AL Rastall RA. Prebiotics and resistance to gastrointestinal infections. Br J Nutr (2005) 93:S31–4. doi: 10.1079/BJN20041343 15877892

[182] Louis P Flint HJ Michel C. How to manipulate the microbiota: Prebiotics. Adv Exp Med Biol (2016) 902:119–42. doi: 10.1007/978-3-319-31248-4_9 27161355

[183] Shokryazdan P Faseleh Jahromi M Navidshad B Liang JB. Effects of prebiotics on immune system and cytokine expression. Med Microbiol Immunol (Berl) (2017) 206:1–9. doi: 10.1007/s00430-016-0481-y 27704207

[184] Ashaolu TJ. Immune boosting functional foods and their mechanisms: A critical evaluation of probiotics and prebiotics. BioMed Pharmacother (2020) 130:110625. doi: 10.1016/j.biopha.2020.110625 32795926

[185] Roberfroid M Gibson GR Hoyles L McCartney AL Rastall R Rowland I. Prebiotic effects: Metabolic and health benefits. Br J Nutr (2010) 104:S1–S63. doi: 10.1017/S0007114510003363 20920376

[186] Peredo-Lovillo A Romero-Luna HE Jimenez-Fernandez M. Health promoting microbial metabolites produced by gut microbiota after prebiotics metabolism. Food Res Int (2020) 136:109473. doi: 10.1016/j.foodres.2020.109473 32846558

[187] Yahfoufi N Mallet J Graham E Matar C. Role of probiotics and prebiotics in immunomodulation. Curr Opin Food Sci (2018) 20:82–91. doi: 10.1016/j.cofs.2018.04.006

[188] Pujari R Banerjee G. Impact of prebiotics on immune response: From the bench to the clinic. Immunol Cell Biol (2021) 99:255–73. doi: 10.1111/imcb.12409 32996638

[189] Liu L Chen D Yu B Yin H Huang Z Luo Y. Fructooligosaccharides improve growth performance and intestinal epithelium function in weaned pigs exposed to enterotoxigenic. Escherichia coli. Food Funct (2020) 11:9599–612. doi: 10.1039/D0FO01998D 33151222

[190] Luo Y Liu L Chen D Yu B Zheng P Mao X. Dietary supplementation of fructooligosaccharides alleviates enterotoxigenic *E. coli*-induced disruption of intestinal epithelium in a weaned piglet model. Br J Nutr (2021), 1–27. doi: 10.1017/S0007114521004451 34763738

[191] Barile D Rastall RA. Human milk and related oligosaccharides as prebiotics. Curr Opin Biotechnol (2013) 24:214–9. doi: 10.1016/j.copbio.2013.01.008 23434179

[192] Sarabia-Sainz HM Armenta-Ruiz C Sarabia-Sainz JA Guzman-Partida AM Ledesma-Osuna AI Vazquez-Moreno L. Adhesion of enterotoxigenic *Escherichia coli* strains to neoglycans synthesised with prebiotic galactooligosaccharides. Food Chem (2013) 141:2727–34. doi: 10.1016/j.foodchem.2013.05.040 23871017

[193] Zhu F Du B Xu B. A critical review on production and industrial applications of beta-glucans. Food Hydrocoll (2016) 52:275–88. doi: 10.1016/j.foodhyd.2015.07.003

[194] Stuyven E Cox E Vancaeneghem S Arnouts S Deprez P Goddeeris BM. Effect of β-glucans on an ETEC infection in piglets. Vet Immunol Immunopathol (2009) 128:60–6. doi: 10.1016/j.vetimm.2008.10.311 19046775

[195] Goodridge HS Wolf AJ Underhill DM. β-glucan recognition by the innate immune system. Immunol Rev (2009) 230:38–50. doi: 10.1111/j.1600-065X.2009.00793.x 19594628 PMC6618291

[196] Dillard CJ German JB. Phytochemicals: Nutraceuticals and human health. J Sci Food Agric (2000) 80:1744–56. doi: 10.1002/1097-0010(20000915)80:12<1744::AID-JSFA725>3.0.CO;2-W

[197] Lee MT Lin WC Yu B Lee TT. Antioxidant capacity of phytochemicals and their potential effects on oxidative status in animals–a review. Asian-Australas J Anim Sci (2017) 30:299–308. doi: 10.5713/ajas.16.0438 27660026 PMC5337908

[198] Zhu F Du B Xu B. Anti-inflammatory effects of phytochemicals from fruits, vegetables, and food legumes: A review. Crit Rev Food Sci Nutr (2018) 58:1260–70. doi: 10.1080/10408398.2016.1251390 28605204

[199] Khameneh B Iranshahy M Soheili V Fazly Bazzaz BS. Review on plant antimicrobials: A mechanistic viewpoint. Antimicrob Resist Infect Control (2019) 8:118. doi: 10.1186/s13756-019-0559-6 31346459 PMC6636059

[200] Ben-Shabat S Yarmolinsky L Porat D Dahan A. Antiviral effect of phytochemicals from medicinal plants: Applications and drug delivery strategies. Drug Delivery Transl Res (2020) 10:354–67. doi: 10.1007/s13346-019-00691-6 PMC7097340 31788762

[201] Bobis O Dezmirean DS Tomos L Chirila F Al. Marghitas L. Influence of phytochemical profile on antibacterial activity of different medicinal plants against gram-positive and gram-negative bacteria. Appl Biochem Microbiol (2015) 51:113–8. doi: 10.1134/S0003683815010044

[202] Borges A J Saavedra M Simoes M. Insights on antimicrobial resistance, niofilms and the use of phytochemicals as new antimicrobial agents. Curr Med Chem (2015) 22:2590–614. doi: 10.2174/0929867322666150530210522 26028341

[203] Barbieri R Coppo E Marchese A Daglia M Sobarzo-Sanchez E Nabavi SF. Phytochemicals for human disease: An update on plant-derived compounds antibacterial activity. Microbiol Res (2017) 196:44–68. doi: 10.1016/j.micres.2016.12.003 28164790

[204] Nazzaro F Fratianni F De Martino L Coppola R De Feo V. Effect of essential oils on pathogenic bacteria. Pharmaceuticals (2013) 6:1451–74. doi: 10.3390/ph6121451 PMC3873673 24287491

[205] Swamy MK Akhtar MS Sinniah UR. Antimicrobial properties of plant essential oils against human pathogens and their mode of action: An updated review. Evid. Based Complement Alternat Med (2016) 2016:e3012462. doi: 10.1155/2016/3012462 PMC5206475 28090211

[206] Seukep AJ Kuete V Nahar L Sarker SD Guo M. Plant-derived secondary metabolites as the main source of efflux pump inhibitors and methods for identification. J Pharm Anal (2020) 10:277–90. doi: 10.1016/j.jpha.2019.11.002 PMC7474127 32923005

[207] Soliman SSM Saeed BQ Elseginy SA Al-Marzooq F Ahmady IM El-Keblawy AA. Critical discovery and synthesis of novel antibacterial and resistance-modifying agents inspired by plant phytochemical defense mechanisms. Chem Biol Interact (2021) 333:109318. doi: 10.1016/j.cbi.2020.109318 33186599

[208] Burt SA van der Zee R Koets AP de Graaff AM van Knapen F Gaastra W. Carvacrol induces heat shock protein 60 and inhibits synthesis of flagellin in *Escherichia coli* O157:H7. Appl Environ Microbiol (2007) 73:4484–90. doi: 10.1128/AEM.00340-07 PMC1932834 17526792

[209] Klancnik A Simunovic K Sternisa M Ramic D Smole Mozina S Bucar F. Anti-adhesion activity of phytochemicals to prevent *Campylobacter jejuni* biofilm formation on abiotic surfaces. Phytochem Rev (2021) 20:55–84. doi: 10.1007/s11101-020-09669-6

[210] Monte J Abreu AC Borges A Simões LC Simoes M. Antimicrobial activity of selected phytochemicals against *Escherichia coli* and *Staphylococcus aureus* and their niofilms. Pathogens (2014) 3:473–98. doi: 10.3390/pathogens3020473 PMC4243457 25437810

[211] Gupta P Song B Neto C A. Camesano T. Atomic force microscopy-guided fractionation reveals the influence of cranberry phytochemicals on adhesion of *Escherichia coli* . Food Funct (2016) 7:2655–66. doi: 10.1039/C6FO00109B 27220364

[212] Joseph SV Edirisinghe I Burton-Freeman BM. Fruit polyphenols: A review of anti-inflammatory effects in humans. Crit Rev Food Sci Nutr (2016) 56:419–44. doi: 10.1080/10408398.2013.767221 25616409

[213] Serafini M Peluso I. Functional foods for health: The interrelated antioxidant and anti-inflammatory role of fruits, vegetables, herbs, spices and cocoa in humans. Curr Pharm Des (2016) 22:6701–15. doi: 10.2174/1381612823666161123094235 PMC5427773 27881064

[214] Miguel MG. Antioxidant and anti-inflammatory activities of essential oils: A short review. Molecules (2010) 15:9252–87. doi: 10.3390/molecules15129252 PMC6259136 21160452

[215] Raut JS Karuppayil SM. A status review on the medicinal properties of essential oils. Ind Crops Prod. (2014) 62:250–64. doi: 10.1016/j.indcrop.2014.05.055

[216] El-Hack MEA Alagawany M Ragab Farag M Tiwari R Karthik K Dhama K. Beneficial impacts of thymol essential oil on health and production of animals, fish and poultry: a review. J Essent Oil Res (2016) 28:365–82. doi: 10.1080/10412905.2016.1153002

[217] Nehme R Andres S Pereira RB Ben Jemaa M Bouhallab S Ceciliani F. Essential oils in livestock: From health to food quality. Antioxidants (2021) 10:330. doi: 10.3390/antiox10020330 33672283 PMC7926721

[218] Surh Y-J Chun K-S Cha H-H Han SS Keum Y-S Park K-K. Molecular mechanisms underlying chemopreventive activities of anti-inflammatory phytochemicals: Down-regulation of COX-2 and iNOS through suppression of NF-κB activation. Mutat Res Mol Mech Mutagen (2001) 480–481:243–68. doi: 10.1016/S0027-5107(01)00183-X 11506818

[219] Saleh HA Yousef MH Abdelnaser A. The anti-inflammatory properties of phytochemicals and their effects on epigenetic mechanisms involved in TLR4/NF-κB-Mediated inflammation. Front Immunol (2021) 12:606069. doi: 10.3389/fimmu.2021.606069 33868227 PMC8044831

[220] Murakami A Ohigashi H. Targeting NOX, INOS and COX-2 in inflammatory cells: Chemoprevention using food phytochemicals. Int J Cancer (2007) 121:2357–63. doi: 10.1002/ijc.23161 17893865

[221] Gomes A Fernandes E Lima JLFC Mira L Corvo ML. Molecular mechanisms of anti-inflammatory activity mediated by flavonoids. Curr Med Chem (2008) 15:1586–605. doi: 10.2174/092986708784911579 18673226

[222] Kumar S Egbuna C. Phytochemistry: An in-silico and in-vitro update: Advances in phytochemical research Vol. . p. . Singapore: Springer Singapore (2019) p. 133–60. doi: 10.1007/978-981-13-6920-9_8

[223] Mohana Devi S Lee SI Kim LH. Effect of phytogenics on growth performance, fecal score, blood profiles, fecal noxious gas emission, digestibility, and intestinal morphology of weanling pigs challenged with *Escherichia coli* K88. Pol J Vet Sci (2015) 18:557–63. doi: 10.1515/pjvs-2015-0072 26618588

[224] Girard M Hu D Pradervand N Neuenschwander S Bee G. Chestnut extract but not sodium salicylate decreases the severity of diarrhea and enterotoxigenic *Escherichia coli* F4 shedding in artificially infected piglets. PloS One (2020) 15:e0214267. doi: 10.1371/journal.pone.0214267 32106264 PMC7046202

[225] Coddens A Loos M Vanrompay D Remon JP Cox E. Cranberry extract inhibits *in vitro* adhesion of F4 and F18+ *Escherichia coli* to pig intestinal epithelium and reduces *in vivo* excretion of pigs orally challenged with F18+ verotoxigenic e. coli. Vet Microbiol (2017) 202:64–71. doi: 10.1016/j.vetmic.2017.01.019 28161211

[226] Ellison R Giehl TJ. Killing of gram-negative bacteria by lactoferrin and lysozyme. J Clin Invest (1991) 88:1080–91. doi: 10.1172/JCI115407 PMC295557 1918365

[227] Ogundele MO. A novel anti-inflammatory activity of lysozyme: Modulation of serum complement activation. Mediators Inflammation (1998) 7:363–5. doi: 10.1080/09629359890893 PMC1781862 9883972

[228] Carrillo W Spindola H Ramos M Recio I Carvalho JE. Anti-inflammatory and anti-nociceptive activities of native and modified hen egg white lysozyme. J Med Food (2016) 19:978–82. doi: 10.1089/jmf.2015.0141 27681299

[229] Ragland SA Criss AK. From bacterial killing to immune modulation: Recent insights into the functions of lysozyme. PloS Pathog (2017) 13:e1006512. doi: 10.1371/journal.ppat.1006512 28934357 PMC5608400

[230] Li YM Tan AX Vlassara H. Antibacterial activity of lysozyme and lactoferrin is inhibited by binding of advanced glycation-modified proteins to a conserved motif. Nat Med (1995) 1:1057–61. doi: 10.1038/nm1095-1057 7489363

[231] Oliver WT Wells JE. Lysozyme as an alternative to antibiotics improves growth performance and small intestinal morphology in nursery pigs1. J Anim Sci (2013) 91:3129–36. doi: 10.2527/jas.2012-5782 23572262

[232] Garas LC Cooper CA Dawson MW Wang J-L Murray JD Maga EA. Young pigs consuming lysozyme transgenic goat milk are protected from clinical symptoms of enterotoxigenic escherichia coli infection. J Nutr (2017) 147:2050–9. doi: 10.3945/jn.117.251322 28954839

[233] Huang G Li X Lu D Liu S Suo X Li Q. Lysozyme improves gut performance and protects against enterotoxigenic *Escherichia coli* infection in neonatal piglets. Vet Res (2018) 49:20. doi: 10.1186/s13567-018-0511-4219 29463305 PMC5819691

